# The Role of Ghrelin/GHS-R1A Signaling in Nonalcohol Drug Addictions

**DOI:** 10.3390/ijms23020761

**Published:** 2022-01-11

**Authors:** Magdalena Sustkova-Fiserova, Chrysostomos Charalambous, Anna Khryakova, Alina Certilina, Marek Lapka, Romana Šlamberová

**Affiliations:** 1 Department of Pharmacology, Third Faculty of Medicine, Charles University, Ruska 87, 100 00 Prague, Czech Republic; chrysostomos.charalambous@lf3.cuni.cz (C.C.); chrjakova.anna@seznam.cz (A.K.); alina7@email.cz (A.C.); marek.lapka@centrum.cz (M.L.); 2 Department of Physiology, Third Faculty of Medicine, Charles University, Ke Karlovu 4, 120 00 Prague, Czech Republic; romana.slamberova@lf3.cuni.cz

**Keywords:** ghrelin signaling, growth hormone secretagogue receptor type A (GHS-R1A), addiction, nicotine/tobacco, stimulants, opioids, cannabinoids, preclinical and clinical research, review

## Abstract

Drug addiction causes constant serious health, social, and economic burden within the human society. The current drug dependence pharmacotherapies, particularly relapse prevention, remain limited, unsatisfactory, unreliable for opioids and tobacco, and even symptomatic for stimulants and cannabinoids, thus, new more effective treatment strategies are researched. The antagonism of the growth hormone secretagogue receptor type A (GHS-R1A) has been recently proposed as a novel alcohol addiction treatment strategy, and it has been intensively studied in experimental models of other addictive drugs, such as nicotine, stimulants, opioids and cannabinoids. The role of ghrelin signaling in these drugs effects has also been investigated. The present review aims to provide a comprehensive overview of preclinical and clinical studies focused on ghrelin’s/GHS-R1A possible involvement in these nonalcohol addictive drugs reinforcing effects and addiction. Although the investigation is still in its early stage, majority of the existing reviewed experimental results from rodents with the addition of few human studies, that searched correlations between the genetic variations of the ghrelin signaling or the ghrelin blood content with the addictive drugs effects, have indicated the importance of the ghrelin’s/GHS-R1As involvement in the nonalcohol abused drugs pro-addictive effects. Further research is necessary to elucidate the exact involved mechanisms and to verify the future potential utilization and safety of the GHS-R1A antagonism use for these drug addiction therapies, particularly for reducing the risk of relapse.

## 1. Introduction

Within the human society worldwide, substance use disorders, also known as drug addiction, cause serious health, social and economic burden on a long-term basis. Europe ranks first in alcohol and tobacco annual consumptions and attributable mortalities; according to an expert estimation, ~5.5 % of all annual deaths in Europe are alcohol-attributable and ~16% of all deaths in adults over 30 are tobacco-related, with many of these deaths occurring prematurely [1,2]. On the contrary, regarding drug overdose deaths involving substances other than alcohol and tobacco, the USA overtakes the European rates by almost 10-times, with 217 drug overdose deaths per million in the population aged 15–64 compared to 22.6 overdose deaths per million (based on data from 2017, though this trend seems enduring). Recently, the majority of the drug overdoses have been caused by opioids [3,4]. An increasing number of fatal overdoses and related deaths in Europe involving cocaine and amphetamines, synthetic cannabinoids, and fake medicines, such as benzodiazepines have also been reported. Cannabinoids have traditionally been the most widely used illegal drugs in Europe and elsewhere, and the effects of novel synthetic cannabinoids are often more severe than those of cannabis. However, in most overdose and drug-related cases, multiple drug toxicity is implicated [3,4].

Drug addiction dramatically aggravates overall harm and increases the fatal consequences of drug use. The drug intake is determined by drug’s pharmacological rewarding effects and is influenced by many internal (genetics, developmental, co-morbidities etc.) and external (social surroundings, stress, drug availability, social norms and systems etc.) factors. Drug addiction is a chronic relapsing brain disease triggered by repeated drug exposures in individuals who are vulnerable due to genetic predispositions and developmental or adverse social exposures [5,6]. The reinforcing effects of drugs mainly depend on the activation of the mesolimbic dopaminergic system, a crucial part of the brain reward system, which is a substantial component of cardinal human survival strategies. Natural survival-strengthening stimuli, such as food and sex, are accentuated by simultaneous pleasurable feelings linked with dopamine release in the nucleus accumbens (NAC) [7,8,9]. This triggers conditioning with the reward originators and influences motivation, control, and decision-making processing, with participation of the hippocampus, prefrontal cortex and other brain areas, including the amygdala. All currently known drugs of abuse induce the massive release of dopamine in the NAC (shell) through direct or indirect pharmacological effects on the dopaminergic neurons in the ventral tegmental area (VTA) and/or the nucleus accumbens (NAC) [5,6,8,10,11,12,13]. Converging evidence indicates that the excitatory cholinergic terminals in the VTA that arise from the laterodorsal tegmental area (LDTg) synapse on the VTA-dopaminergic neurons, which preferentially innervate the NAC [14]. The cholinergic-dopaminergic circuitry possibly contributes to the net rewarding effects, mandates motivational behaviors, and may participate in drug reinforcement [15,16,17,18,19]. Additional neurotransmitter systems contribute to the drug-induced reinforcing effects, including dopamine-modulating or dopamine-independent mechanisms, e.g., endogenous opioids, endocannabinoids, gamma-aminobutyric acid (GABA), acetylcholine, serotonin, and neuropeptides [20,21,22,23,24,25,26]. The unnaturally excessive drug-induced activation of the pre-set reward/reinforcement system, consequently overburdens the interconnected brain circuits, including the decision- and control-regulating systems. In vulnerable individuals, repeated/chronic drug exposure leads to drug-centric behavior, self-regulation impairment, and addiction.

There is a noticeable overlap of involved neuronal systems [27] as well as a high comorbidity between eating disorders and substance use disorders [28]. The prevalence of substance use was found to be increased with food restriction/dieting severity [29], and food restriction was found to enhance the central rewarding effects of abused drugs [30,31,32], which inspired the investigation into the involvement of appetite regulating substances in drug addiction. Recent studies have already implicated several neuropeptides of the gut-brain axis, such as ghrelin, glucagon-like peptide/GLP-1, amylin, and neuromedin U/NMU, as modulators of reward and proaddictive processes [26]. The only orexigenic gut-brain peptide hormone, ghrelin, commonly called “hunger hormone”, has been extensively studied for more than 20 years since its discovery [33]. It has been documented that ghrelin exerts its potent orexigenic and pro-obesity effects not only through metabolic homeostatic regulatory mechanisms in the hypothalamus, but also by significant increasing food reward and motivation through mesolimbic activation [34,35,36]. Ghrelin predominantly arises from gastric endocrine mucosal cells and is mainly released by an empty stomach [37,38,39]. Small formations of ghrelin have also been found in several peripheral organs (testis, ovary, placenta, kidney, small intestine, pancreas, lymphocytes etc.) as well as in the brain (hypothalamus) [40,41,42]. The notion that ghrelin can be produced in the central nervous system of mammals in physiologically relevant levels still remains controversial [43]. Ghrelin is cleaved from the preproghrelin peptide, which is encoded by the GHRL gene on chromosome 3. Ghrelin, when modified by the ghrelin-O-acyl transferase (GOAT), forms acyl-ghrelin. This n-octanoyl form of ghrelin, acylated at serine 3, activates the growth hormone secretagogue receptor type A1 (GHS-R1A) and releases the growth hormone [33,38]. Thus, acyl-ghrelin is called the active form of ghrelin. The des-acyl ghrelin (without the n-octanoyl moiety) represents the majority form of ghrelin in the blood and behaves as a separate hormone with a number of physiological functions [44,45]. It is mostly inactive at the GHS-R1A but can variously influence the acyl-ghrelin effects [38,45,46,47,48]. Both ghrelin forms are detectable in the blood circulation and can pass through the blood brain barrier to the brain [49,50]. Some studies have indicated that peripheral ghrelin administration might not directly reach the deep brain areas other than the hypothalamus [51,52,53,54]; therefore, it was proposed that alternative indirect mechanisms of the observed peripheral ghrelin effects could also be considered in the mesolimbic areas (e.g., through cholinergic neurons or orexin-containing neurons projecting from hypothalamus to these reward-related areas) [26,55]. Additionally, acyl-ghrelin independent central effects on the ghrelin receptors have been suggested (see below).

Acyl-ghrelin mediates its plentiful peripheral as well as central physiological functions through the GHS-R1A, which is encoded by the GHSR gene on chromosome 3 [56,57]. Through the GHS-R1A, the acylated ghrelin modulates systemic metabolism, food intake, and various other peripheral and central physiological functions, such as inflammation, thermogenesis, cardiac output and contractility, the stimulation of gastric motility, prolactin secretion, depression, sleep-wake rhythm, neuroprotection, memory, and reward behavior [38,58,59,60]. Within the brain, in addition to the hypothalamus, the GHSR1As are expressed in important reward related areas including the striatum, NAC, amygdala, prefrontal cortex, hippocampus, and VTA [35,42,56,61,62,63,64,65,66]. The GHS-R1A is metabotropic and uses several different G-protein secondary-messenger signaling pathways [66,67]. The GHS-R1A also shows high constitutive activity, i.e., intrinsic activity that is independent of ligand binding, that can be reduced by treatment with inverse agonists [58,68,69,70,71]. It is assumed that the ligand-independent activity is also important for several central processes, e.g., reward, memory, and anxiety [54,71]. GHS-R1A oligomerizations and dimerizations have been found with a wide array of other G-protein coupled receptors, including dopamine receptors D1R and D2R, the serotonin 2C receptor (5-HT-2C), and the melanocortin (MC3R, MC4R), somatostatin, oxytocin, and sigma-1 receptors [72,73,74,75]. It was suggested that the GHS-R1A might also form a heterodimer with the cannabinoid CB1 receptor (CB1R) [72]. Furthermore, another variant of the ghrelin receptor, GHS-R1B, forms heterodimers/oligomeric complexes with the GHS-R1A and acts as a dual modulator of its functions [58,76]. In actual fact, (acyl-)ghrelin does not bind to the GHS-R1B; nevertheless, this receptor subtype not only determines the efficacy of ghrelin-induced, GHS-R1A-mediated signaling but also seems to determine the ability of GHS-R1A to form oligomeric complexes with other receptors (e.g., D1R), promoting profound qualitative changes in ghrelin-induced signaling [47,76,77]. However, the functional role of GHS-R1B still remains much less explored [74,78,79]. Recently, the liver-expressed antimicrobial peptide 2 (LEAP2) was described as an endogenous antagonist/inverse agonist of the GHS-R1A which regulates the transmission of intracellular signals from ghrelin [58,80,81].

The strong constitutive activity of the GHS-R1A and its aforementioned ability to heterodimerize with the D1R/D2R in the VTA possibly alter the sensitivity of the mesolimbic dopamine system. Central GHS-R1A receptors are frequently colocalized with dopaminergic and cholinergic receptors [42,57], and it is believed that midbrain functional interactions between these receptors amplify the dopaminergic signaling in the VTA neurons and stimulate the overflow of dopamine in the NAC [76,82,83]. The systemic and central (into the VTA) administration of acyl-ghrelin induced the dopamine release in the NAC (shell) and locomotor hyperactivity in rodents [61,64,82,84,85]. The injection of acyl-ghrelin into the LDTg, where the GHS-R1As are located on the cholinergic neurons, also stimulated the locomotor activity, caused accumbens dopamine efflux, and concomitantly enhanced the levels of the VTA-acetylcholine via the nicotinic cholinergic receptor (nAChR)-dependent mechanism [55,84]. Additionally, the peripheral injection of acyl-ghrelin induced the concomitant release of VTA acetylcholine and accumbens dopamine [55]. These findings suggest that ghrelin stimulates the cholinergic-dopaminergic circuitry, possibly via the nAChR in the VTA, and as previously mentioned, this circuitry is involved in motivational and reward behaviors [18,19]. Thus, ghrelin/GHS-R1A play a crucial role in general reward processing.

Acyl-ghrelin also interacts with the hypothalamic-pituitary-adrenal axis (HPA) and modulates stress-related behaviors [86,87]. On the basis of animal studies, it appears that ghrelin might exert anxiolytic effects in acute stress conditions, while promoting anxiogenic behavior in unstressed and chronically stressed conditions [87,88,89,90,91]. The apparent roles of ghrelin in reward- and stress-related systems suggested the importance of its participation in addiction processes, including drug addiction, which are typically associated with positive (reward) and negative (stress relieve) reinforcements [5,6,82,87]. Indeed, an increasing number of experimental and clinical studies have started to investigate the possible associations between drugs of abuse and ghrelin signaling.

Initial preclinical experiments explored and evidenced the ghrelin’s involvement in the processing of reinforcing effects of alcohol, which can be considered both a palatable food and energy resource and an addictive substance [92]. The relationships between ghrelin/GHS-R1A and alcohol have been extensively studied—see the several recent comprehensive reviews [26,93,94,95]. Based on a significant number of encouraging preclinical results, ghrelin/GHS-R1A antagonism was suggested as a novel treatment approach for alcohol addiction therapy and recently, the first GHS-R1A-related compound was tested in initial human laboratory studies. The GHS-R1A-inverse agonist (PF5190457) was found to be well-tolerated and safe in healthy volunteers [96], and in a single-blind, within-subject, placebo controlled human laboratory study in heavy alcohol drinkers, it was found to significantly reduce the alcohol cue-induced craving in comparison to the placebo, while the safety/tolerability assessment revealed only mild or moderate adverse events, which did not require the discontinuation or dose reduction of the PF5190457 [71,97]. A robust tow-compartment model describing the pharmacokinetic characteristics of PF5190457 has been created [98]. Further research continues.

Gradually, the possible relationships between ghrelin/GHS-R1As and several other drugs of abuse have also been explored, and many interesting results have already been assembled in several reviews [26,99,100,101]. Recent investigation on ghrelin signaling and nonalcohol addictive drugs interactions possibly provided the most novel contribution, and therefore our article focuses on this topic. As previously mentioned, the most advanced and extensive research of alcohol-ghrelin relationships, including an initial clinical research of the potential utilization of ghrelin antagonism for alcohol addiction therapy, has been described in-detail in complex, up-to-date reviews [26,93]. The update of the clinical study using PF5190457 was mentioned above [71,97,98]. Thus, the current review summarizes essential existing preclinical and clinical studies on the interaction between ghrelin/GHS-R1A system and nonalcohol drugs of abuse, specifically nicotine, stimulants, opioids, and cannabinoids.

Drug dependence research uses experimental addiction models. The most important methods that are mentioned in the review are briefly summarized below. The administration of addictive drugs typically induces behavioral stimulation/hyperlocomotion in rodents, involving stereotypical behaviors (licking, confined gnowing, sterotyped sniffing, etc.), which is considered to be a sign of nigrostriatal dopaminergic pathway activation [6,19]. Furthermore, behavioral sensitization (associated with accumbens dopaminergic sensitization), which is caused by repeated addictive drug administration, is considered within certain limits to be a manifestation of the incentive motivation of underlying drug-seeking behavior [18,102,103]. Incidentally, the subchronic systemic ghrelin treatment itself has also induced locomotor sensitization [104], which corresponds with ghrelin’s reinforcement ability. The drug-conditioned place preference (CPP) method is mainly focused on association/conditioning of environmental cues with a substance effect, which plays an important role in the acquisition and maintenance of addiction [105,106,107]. A crucial experimental addiction model, intravenous self-administration (IVSA), is used to assess the rewarding and reinforcing effects of drugs and to evaluate the principle treatment goal of abolishing/reducing drug consumption [107]. A specific IVSA arrangement with a period of abstinence allows the assessment of the tendency to relapse, i.e., drug-seeking behavior [108,109]. Ghrelin signaling and blood levels are affected by many factors [110], including satiety/hunger status. Circulating ghrelin is enhanced following hunger/food deprivation [38,39,111,112]; thus, the food conditions might have impacted some of the results mentioned in this review. In the majority of the experimental studies reviewed in this paper that tested the effects of addictive drugs, the animals had free access to standard food, which usually was removed during testing (unless otherwise specified). In clinical studies, the food conditions were more heterogenous.

In this review, we summarize our current knowledge from preclinical and clinical studies within the ghrelin/nonalcohol drugs of abuse research, highlight the imperfections, and suggest possible directions for future research.

## 2. Ghrelin/GHS-R1As and Nicotine/Tobacco

Tobacco leaves (Nicotiana plant) contain psychoactive and addictive alkaloid nicotine and have been broadly used for centuries (smoked; chewed; snorted; and, recently, vaped). Despite the generally agreed-upon harmful consequences of tobacco use and official public health antismoking interventions, in 2021 around 23% of the European population were estimated to be current tobacco smokers [113,114]. Furthermore, the novel phenomenon of electronic cigarettes use is not without its risks [115]. Tobacco/nicotine is potentially addictive in all its forms of use [116]. Nicotine activates the brain reward system directly through the cholinergic nicotine receptors on the dopamine neurons in the VTA and also indirectly via the glutamate and GABA modulatory effects [117,118,119]. Tobacco/nicotine influences appetitive signals, reduces food intake and body weight gain [120]; thus, the relationship between ghrelin and nicotine in food intake has been explored. In food-deprived women, it was found that after a caloric load, nicotine amplified the positive correlation between fasting ghrelin levels and changes in food-cue reactivity in the brain, particularly in the medial prefrontal cortex (mPFC) and amygdala, which might contribute to nicotine’s anorectic potential (alongside the negative correlations with augmented leptin) [121]. Nicotine intake/cigarette smoking increased among women during acute food deprivation [122]; thus, the role of ghrelin in nicotine’s proaddictive effects has also been investigated in a whole range of preclinical and clinical studies.

### 2.1. Preclinical Studies

Several rodent studies have explored the tobacco/nicotine effects on ghrelin blood levels. The prolonged exposure of rats to cigarettes smoke (5 days weekly for a total of four weeks) significantly augmented the acyl-ghrelin blood levels, while the des-acyl ghrelin concentration remained unaffected [123]. In another study, four weeks of cigarette smoke exposure did not significantly affected the total ghrelin blood levels in rats [124], which might correspond with the predominant proportion of the des-acyl ghrelin in the total ghrelin amount [38]. The three-week administration of nicotine in rats (oral, inhaled, intraperitoneal) resulted in a significant increase in the serum and gastric homogenate ghrelin concentrations [125]; the authors did not state the detected ghrelin type; however, the results indicate that the content of total ghrelin was analysed [125].

In vitro experiment using rat brain slices revealed that acyl-ghrelin amplified the nicotine-induced dopamine release in the striatum [85]. Interestingly, the acyl-ghrelin-induced striatal and also amygdalar dopamine release in vitro was inhibited by both the selective GHS-R1A antagonist (D-Lys-3-GHRP-6) and selective nicotinic nAChR antagonist (mecamylamine), which suggests that the striatal cholinergic interneurons also express GHS-R1A [85,126] and that the nicotinic nAChRs are significantly involved in mediating the dopamine-enhancing/reinforcing effect of ghrelin, in accordance with previous findings in mice [82].

The pharmacological ghrelin/GHS-R1A antagonism induced by pretreatment with a single systemic JMV2959 [127] dose significantly reduced intraperitoneal nicotine-induced hyperlocomotion, conditioned place preference (CPP), and accumbens dopamine release in mice [128]. JMV2959 pretreatment also attenuated repeated nicotine-induced locomotor sensitization in rats [129]. Ghrelin antagonism has not yet been tested in any nicotine/tobacco self-administration studies.

### 2.2. Clinical Studies

The existing human studies searched for associations between ghrelin/GHS-R1A genetic varieties and ghrelin (blood) content changes and nicotine/tobacco use. A human genetic study that was focused on female patients with severe alcohol dependence in Sweden reported that two haploid genotypes of the GHSR gene were associated with smoking status [130]. Another study showed that the minor allele of the rs2948694 single-nucleotide polymorphism (SNP) was located in the GHSR-1A gene (GHSR), but none of the tested SNPs of the pre-proghrelin gene (GHRL) appeared to be associated with an increased severity of cigarette smoking [131].

Further human studies have sought associations between ghrelin blood levels and tobacco/nicotine use and/or withdrawal. Although acyl-ghrelin is considered to be the active GHS-R1A natural agonist, more stable total ghrelin levels were predominantly measured. The acute smoking of one cigarette increased the fasting total ghrelin levels in both smokers and non-smokers in a small study [132]. However, another study described no immediate effect on the fasting total ghrelin levels of smoking two consecutive cigarettes in habitual smokers and suggested a decrease in the total ghrelin in non-smokers [133]. Another study in healthy non-smokers found that the total ghrelin levels were not affected by acute cigarette smoking, but that the ghrelin content was significantly reduced in saliva (satiety status not mentioned) [134]. Two studies have noted that chewing nicotine gum in healthy non-smokers did not significantly affect the blood total/des-acyl ghrelin levels (fasting ghrelin [121]; satiety status not mentioned [135]). The variability of results might be partly explained by some methodological inconsistencies (e.g., different satiety status during blood sampling or other factors—see the discussion in Section 6).

Series of human studies have measured the ghrelin levels in habituated tobacco smokers; however, these are frequently only the secondary outcome measurements of studies with various aims in different research areas (metabolic, endocrinologic, cardiovascular, etc.), and therefore contain substantial methodological variabilities. Some of these studies found no correlation between the total ghrelin levels and tobacco smoking (pregnant women, fasting ghrelin [136]; fasting ghrelin [133]; large study in Finnish population, total 1024 subjects with atherosclerosis, satiety status not mentioned [137]) One study noted that the plasma concentration of acetylated ghrelin, but not the total ghrelin, was significantly higher in smokers than in non-smokers and that lower acyl-ghrelin levels were correlated with the severity of nicotine dependence (blood sampled 2 h after meal and 3h after last cigarette) [138]. By contrast, in several studies, increased fasting total ghrelin blood levels were observed in current smokers in comparison to nonsmokers [139,140], including an extensive recent population-based cohort study (“LIFE-Adult”) in the German population (total 1519 subjects) [141], where the overnight-fasting total ghrelin levels were positively associated with active, but not former, tobacco smoking. Some studies showed that total ghrelin levels positively correlated with length of a person’s smoking history and may predict the risk of smoking relapse (blood sampled 2 h after meal) [142,143]. However, ghrelin levels were not associated either with the amount of tobacco consumed or with craving in active smokers (blood sampled 2 h after meal [142]; fasting ghrelin [141]). Another small study showed significant decreases in fasting acyl-ghrelin levels during abstinence from tobacco in comparison to the levels prior to smoking cessation (Korean population; abstainers from smoking for 2 months) [144]. An interesting novel study (Caucasian participants; satiety status not mentioned) [145] took into consideration the fact that ghrelin also modulates stress responses and examined the relationship between early life adversity (ELA) and circulating total ghrelin during ad libitum smoking and early withdrawal in tobacco smokers who were interested in cessation. Results showed that ELA was positively associated with elevated total ghrelin in non-smokers (in accordance with similar previous findings for acyl-ghrelin in non-smokers [146]). Among those reporting no ELA, successful smoking quitters had higher total ghrelin levels than the non-smokers during ad libitum smoking. In principle, compared to nonsmoking, active smoking seemed to be associated with a total ghrelin blood level increase, which was attenuated by smoking withdrawal.

### 2.3. Summary

The relevant reviewed results are summarized in Table 1. (See also Tables S1 and S2 in the Supplementary Information). The preclinical results suggest the clear involvement of the GHS-R1A in the nicotine-induced dopamine mesolimbic changes and show that GHS-R1A antagonism significantly attenuates nicotine’s reinforcing effects in rodent neurobehavioral models. The impact of tobacco smoke/nicotine on ghrelin blood levels in rodents (usually increase) was indicated with lower consistency. A study on ghrelin and ghrelin antagonism in a nicotine/tobacco self-administration model is missing. Additionally, the human studies focused on finding of possible associations between tobacco smoking and the circulating ghrelin concentrations provided rather inconsistent results. Usually, but not always, active smoking was associated with an increase in total ghrelin and acyl-ghrelin levels, which was attenuated during smoking cessation/withdrawal. Substantial methodological variabilities could have contributed to the heterogeneous results observed, such as the conditions of food intake/fasting regimen, gender, variable smoking grade, unverified abstinence, various comorbidities, possible stress history (ELA), concurrent medications, and different ghrelin content determination procedures. Therefore, future studies with conscientious methodological approaches might improve the comparability of the obtained results. Single nucleotid polymorphism (SNP) in the GHS-R1A gene seems to be associated with severe smoking. In general, current results insinuate that the GHS-R1A might play a more evident role in the pro-addictive effects of tobacco/nicotine than (endogenous) ghrelin itself; however, additional research with thoroughly defined methodology would help to elucidate these relationships. The existing results justify continuing this research, which is necessary to clarify the exact role of ghrelin/GHS-R1A in the proaddictive effects of nicotine/tobacco and to test the potential usability of GHS-R1A antagonism for the prevention and treatment of tobacco/nicotine addiction.

## 3. Ghrelin/GHS-R1As and Stimulants

Psychostimulants include a number of synthetic and natural compounds. The most prevalent and harmful (causing health damage, violence, death, etc.) are traditionally considered to be cocaine, amphetamine, and its derivative methamphetamine. In Europe, nearly 3 million young adults aged 15–35 (2.4% of this age population) are estimated to have used cocaine and about 1.5 million are estimated to have used amphetamines (1.2%) in the past year, and the trend of the stimulant use is increasing [147]. Concerning their pharmacological effects, stimulants directly increase the dopamine concentrations in the NAC, mainly through axonal membrane monoamine-dopamine transporters (MATs) manipulation. Cocaine acts as short-term monoamine reuptake inhibitor [148], while amphetamines promote reverse transport (efflux) through MATs and also induce synaptic vesicle depletion (the blockage of VMATs), thus causing a massive prolonged accumbens dopamine increase [149,150]. Psychostimulants show stimulatory and sympathomimetic effects and induce a loss of appetite [151,152]. Food restriction (FR) increases stimulant (amphetamine, D1 agonist A77636) consumption and signs of reward [30,31], and increases amphetamine/cocaine intake and the reinstatement of drug-seeking behavior [153,154]. Thus, the relationship between ghrelin/GHS-R1As and the pro-addictive effects of stimulants has been explored in a considerable number of preclinical studies and two clinical studies so far.

### 3.1. Preclinical Studies

The results of experiments in rodents suggest that central ghrelin signaling plays an important role in the reinforcing effects of stimulants. The administration of stimulants increased peripheral ghrelin availability. The single intraperitoneal administration of either methamphetamine or 3,4-methylendioxy-methamphetamine (MDMA, “ecstasy”) significantly increased total ghrelin blood levels in rats [155,156]. Another study described a positive correlation between total ghrelin serum levels and increased cue-induced cocaine-seeking activity in rats [157]. A recent elaborated investigation showed that the intravenous self-administration (IVSA) and extinction/anticipation of cocaine in rats significantly increased both acyl- and des-acyl ghrelin blood levels, and the cocaine self-administration experience was associated with enhanced responses of acyl- and des-acyl ghrelin to cocaine [158,159]. Thus, the elevation of ghrelin blood levels by cocaine seems to play a critical role in the maintenance of cocaine IVSA and cocaine-seeking motivated by cocaine-conditioned stimuli. The achievement of cocaine IVSA also significantly up-regulated the GHS-R1A mRNA expression in the rat’s VTA [159]. Furthermore, it was found that blockade of peripheral adrenergic ß1 receptors by hydrophilic atenolol potently attenuated the elevation in circulating ghrelin induced by cocaine [159]; atenolol pretreatment also inhibited the IVSA of cocaine [159]. Stomach ghrelin secretion is modulated by the sympathetic nervous system and is increased by ß-sympathomimetics [160]. The observed adrenergic—ghrelinergic interactions require further study. As previously mentioned, the GHS-R1A interacts with the sigma-1 receptors in CNS neurons [75], and cocaine mediates many of its central effects via the sigma-1 receptors through molecular interactions with dopamine and possibly other metabotropic G-protein coupled receptors. Cocaine increases colocalisation of sigma-1R and GHS-R1A at the cell surface (in vitro), and it was indicated that cocaine’s effects on the GHS-R1A were mediated by the sigma-1R [75]. Thus these relationships should be further investigated. Pretreatment with external acyl-ghrelin before the administration of cocaine significantly augmented the cocaine-induced locomotor hyperactivity [161]. Furthermore, repeated systemic acyl-ghrelin administration before acute cocaine exposure augmented the cocaine-induced hyperlocomotion [162]. Ghrelin’s enhancement of psychostimulants’ functions may be caused by its direct action on the mesolimbic dopamine function or may reflect an indirect action of ghrelin on the glucocorticoid pathways [163,164]. It was suggested that NAC-core (rather than NAC-shell) is the neuronal substrate mediating the expression of behavioral activation and drug-induced sensitization [165,166]. It was found that the microinjection of acyl-ghrelin directly into the NAC core significantly sensitized the cocaine- and amphetamine-induced hyperlocomotion, which was abolished by the co-microinjection of GHS-R1A antagonist (D-Lys3-GHRP6), while microinjected ghrelin alone did not change the rats’ behavior [167,168]. Furthermore, previous subchronic exposure to amphetamine in rats produced behavioral sensitization to the microinjection (NAC-core) of acyl-ghrelin when administered together with dopamine D1 agonist (SKF81297), indicating the important interaction of ghrelin with D1 receptors in sensitized locomotion [168].

Similarly to food restriction, which increased ghrelin blood levels (see above), acyl-ghrelin administration also increased the reinforcing effects of stimulants. Pretreatment with systemic [169] as well as central acyl-ghrelin (into the VTA and/or NAC) [170,171] together with intraperitoneal cocaine during a place-conditioning procedure increased cocaine-conditioned place preference (CPP) development in rats, which was attenuated when GHS-R1A antagonist (JMV2959) was centrally coadministered with ghrelin [171]. Another study indicated that the GHS-R1A’s effects on reward are independent from peripheral acyl-ghrelin binding [54], because the administration of acyl-ghrelin antibodies, which blocked the peripheral acyl-ghrelin access to the CNS, did not influence the development of cocaine-conditioned place preference (CPP) in mice, even though the mice weight gain was significantly blunted. However, both weight gain and cocaine rewarding effects (CPP) were blocked by the direct GHS-R1A antagonist (JMV2959). These results indicate that the central GHS-R1As that regulate weight-gain behavior are physiologically dependent on peripheral acyl-ghrelin activation, whereas hedonic reward due to cocaine administration does not appear to require peripheral acyl-ghrelin [54]. It was further found that ghrelinergic and dopaminergic signaling interact in the neuronal control of motivation with other drug rewards (e.g., ethanol). Low doses of acyl-ghrelin (2.5 nmol intraperitoneally) and also ghrelin microinjection into the VTA augmented cocaine-induced voluntary alcohol consumption in rats; conversely, a high ghrelin dose (10 nmol) suppressed it [172]. This seems in accordance with observations recorded when only high doses of ghrelin (including 10 nmol) elicited anxiogenic responses [173,174].

A number of studies have used the pharmacological or genetic suppression of the GHS-R1A to further support the crucial role of central ghrelin signaling in the stimulant’s reinforcement and motivation effects. Initially, it was noted in mice that pretreatment with the systemic GHS-R1A antagonist (JMV2959) attenuated both intraperitoneal cocaine and methamphetamine-induced hyperlocomotion, the expression of drug-induced CPP, and the accumbens dopamine efflux [175]. Furthermore, sub-chronic GHS-R1A blocking by repeated JMV2959 administration attenuated the ability of acute amphetamine to stimulate locomotion in mice [104], while there was no effect of sub-chronic JMV2959 treatment on locomotor activity per se or on the expression of the GHS-R1A gene in the VTA or the NAC compared with vehicle treatment. In another study, the repeated systemic administration of JMV2959 together with cocaine, as well as genetic ablation of the GHS-R1A, significantly attenuated cocaine-induced locomotor sensitization in rats, which suggests that GHS-R1A activity is required for the induction of locomotor sensitization to cocaine [176]. Furthermore, pretreatment with JMV2959 significantly reduced the expression of methamphetamine-conditioned place preference (CPP) in rats, and the simultaneous repeated administration of JMV2959 with methamphetamine during conditioning significantly reduced methamphetamine-CPP development [177], which indicates, that ghrelin antagonism reduced the methamphetamine reward as well as the signs of craving/desire for methamphetamine.

The first intravenous self-administration (IVSA) study found that the blockading of the GHS-R1A by JMV2959 significantly reduced the spontaneous maintenance methamphetamine intake and reduced or prevented the tendency to relapse, as tested in rats on the 12th day of the abstinence period [177]. A recent innovative and comprehensive study noted that JMV2959 pretreatment dose-dependently inhibited cocaine-IVSA, cocaine-seeking, and reinstatement of cocaine-seeking triggered by cocaine in rats [159]. Furthermore, JMV2959 pretreatment also inhibited the brain stimulation reward (BSR) and cocaine-potentiated BSR maintained by optogenetic stimulation of the VTA dopamine neurons in DAT-Cre mice [159].

A recent microdialysis study in rats explored in detail the mesoaccumbal ghrelin—dopamine interactions participating in the stimulants’ reinforcing effects [178]. Previously, it was suggested that not only the dopamine release in the NAC shell, but the concomitant dopamine release in the VTA and NAC shell might represent a common underlying characteristic induced by various reinforcing stimuli, including natural rewards such as food/water [179], drugs of abuse [12,180,181] and the orexigenic peptide ghrelin (included due to later results) [178]. When acyl-ghrelin was infused into the third ventricle (i.c.v.), the dopamine levels increased in both the (anterior) VTA and the NAC shell, while a systemic pretreatment with the JMV2959 fully blocked the ability of ghrelin (i.c.v.) to increase the dopamine levels in both areas. Furthermore, the dopamine D1 receptor antagonist (SCH23390) administered systemic or centrally into the VTA and NAC shell fully blocked the ability of ghrelin (i.c.v.) to increase locomotor activity in mice [178]. Interestingly, when the authors tested amphetamine’s mesoaccumbal effects, systemic JMV2959 pretreatment reduced (rather than fully blocked) the systemic amphetamine-induced concomitant dopamine release in both the VTA and the NAC shell, without altering dopamine levels per se [178]. These results further indicated that the ghrelin/GHS-R1A signaling significantly modulates the ability of stimulants to activate the mesoaccumbal dopamine pathway, which is associated with their reinforcing effects.

### 3.2. Clinical Studies

Thus far, only two clinical studies have investigated the possible role of genetic variability of ghrelin signaling system in the (meth)amphetamine dependence, the severity of use, and/or the emotional problems associated with the use of the drugs. An initial study [182] showed no association between pre-proghrelin gene (GHRL) variations and susceptibility to the development of methamphetamine dependence in a sample of the Korean population but found a significant correlation between carrying the GHRL single nucleotide (Leu72Met) polymorphism and emotional problems, such as depression or anxiety, which are associated with drug addiction. The second clinical study [183] found that the single nucleotide polymorphism (SNP) located on the ghrelin receptor gene GHSR (rs2948694) seemed to be associated with amphetamine dependence, because it was significantly more common among Swedish amphetamine-dependent individuals when compared to healthy controls. No significant differences were found in the pre-proghrelin gene GHRL SNP between the amphetamine-dependent and healthy populations; however, amphetamine-addicted patients carrying the minor allele of the GHRL SNP (rs4684677) showed significantly higher amphetamine-addiction severity scores (Addiction severity index, ASI).

In several clinical articles, ghrelin blood levels were analysed in connection with stimulant administration under miscellaneous conditions. A small study found no significant effects of intravenous cocaine on total or acyl-ghrelin blood levels in experienced cocaine users (the satiety status not mentioned) [184]. One study measured the ghrelin changes in children with attention deficit hyperactivity disorder (ADHD) before and after two month of treatment with methyphenidate, and the fasting total ghrelin blood levels were significantly increased; no differences were found between the baseline total ghrelin levels of ADHD patients and the corresponding healthy control group [185].

### 3.3. Summary

The relevant reviewed results are summarized in Table 2. (See also Tables S1 and S2 in the Supplementary Information). The existing preclinical experiments in rodents suggest that ghrelin/GHS-R1A signaling is significantly involved in the mesoaccumbal effects of stimulants. Furthermore, acyl-ghrelin administration can enhance several behavioral correlates of stimulant abuse. In addition, GHS-R1A antagonism can attenuate the association between stimulants’ effects and the surroundings (CPP), and it can significantly reduce spontaneous stimulant (methamphetamine, cocaine) intake, drug seeking, and the reinstatement of drug-seeking behavior. Moreover, the possible involvement of ghrelin/GHS-R1A interactions with other neural systems (e.g., the adrenergic system, sigma-1 receptor) in stimulants’ reinforcing effects has been discussed in several studies. Scarce human genetic data indicated an association between a single nucleotide polymorphism in the GHS-R1A gene and amphetamine dependence. Existing human studies have measured ghrelin blood changes in connection with stimulant administration as additional information within research focused on other topics; therefore, further clinical studies aimed specifically at the problem of ghrelin and stimulants are warranted. The involvement of ghrelin/GHS-R1A signaling in stimulant reinforcement proaddictive effects seems to be clearly implied; however, additional preclinical and (mainly) clinical studies are required to further elucidate the relationship between ghrelin and stimulants and to test the relevance of the possible future use of ghrelin/GHS-R1A antagonism in the prevention and treatment of stimulant addiction.

## 4. Ghrelin/GHS-R1A and Opioids

Opioids use is a major contributor to the harm associated with drug use in Europe, the USA and elsewhere [3,4]. High-risk opioid use among adults (aged 15–64) was estimated to affect 1.3 million of the European population in 2018 [147]. Abused opioids include traditional illicit drugs (e.g., heroin), prescription opioids (e.g., painkillers such as morphine, fentanyl, oxycodone, tramadol, codeine) and an increasing number of potent abused “new synthetic opioids” of various structures (e.g., derivatives of fentanyl/phenylpiperidines, benzamides, acetamides, piperazines etc.) [187]. The rewarding reinforcing effects of opioid/µ-receptor agonists are traditionally associated with opioid-induced accumbens dopamine efflux caused by supressing the release of γ-aminobutyric acid (GABA) from the VTA interneurons, which tonically inhibit the mesolimbic dopamine neurons [188,189]. Simultaneous opioid-induced dopamine release in the VTA might contribute to the drugs’ reinforcing effects [190]. Increased mesolimbic GABA concentrations potentiate opioid-induced accumbens dopamine release, and GABA contributes to opioid reinforcing effects [24,191]. Additionally, the endogenous cannabinoid system provably significantly participates in opioid reinforcement processes [192,193,194]. Opioids trigger the release of anandamide (arachidonoylethanolamine AEA) in the NAC shell, which possibly participates in opioid reward in a dopamine-nondependent manner [195]. Similarly to the ghrelin system, the endogenous opioid system is also significantly involved in regulation of response to natural rewards including food, and µ-receptor agonists can increase food motivation and intake [196,197]. Several studies have documented important interactions between ghrelin and opioid signaling (particularly within the VTA) in regulating the mesolimbic dopamine system and food reward/reinforcing effects [198,199]. Furthermore, the interactions between ghrelin and cannabinoid systems in food intake regulation have been described [200] (see the next section). Thus, part of existing preclinical research on the involvement of ghrelin/GHS-R1A signaling in opioid addiction noted in this paper has involved the interactions between the opioid, ghrelin, GABA, and endocannabinoid systems. A single clinical study investigated the ghrelin plasma levels in (only) 28 patients with opioid-use disorders in comparison with healthy controls.

### 4.1. Preclinical Studies

Initial experiments noted, that the μ-receptor antagonist naltrexone did not attenuate ghrelin-induced food intake [35] nor ghrelin-induced locomotor stimulation [83], which indicated, that μ-receptors do not play any role in the ghrelin effects. Initial experiments using heroin IVSA model in rats produced rather incoherent results. In accordance with previous findings, wherein food deprivation reinstated heroin seeking [201,202], the intracerebroventricular (i.c.v.) injection of acyl-ghrelin also increased breakpoints on a progressive ratio schedule of heroin reinforcement, which indicated increased drug-seeking [203]. In contrast, the i.c.v. administration of the GHS-R1A antagonist, D-Lys-3-GHRP-6 had no effect on ongoing heroin self-administration under a fixed-ratio 1 schedule (FR1), or on the food deprivation-induced reinstatement of heroin seeking [203]. Nevertheless, recent experiment of the same research group, wherein JMV2959 was microinjected into the VTA, showed that the GHS-R1A antagonism dose-dependently decreased heroin seeking in the food-restricted rats [204], which confirmed the similar JMV2959 effects published previously elsewhere (see below).

The first microdialysis study with morphine in rats documented, that GHS-R1A antagonist JMV2959 pretreatment significantly and dose-dependently reduced morphine-induced dopamine release in the NAC shell and affected concentration of byproducts associated with dopamine metabolism [205]. Specifically, the accumbens 3,4-dihydroxyphenylacetic acid (DOPAC), and homovanillic acid (HVA) concentrations were significantly increased, which indicated that ghrelin antagonism possibly increased metabolism of dopamine by monoamine oxidase (MAO). JMV2959 pretreatment also significantly reduced morphine-induced behavioral stimulation, especially stereotyped behavior [205]. Corresponding results were later obtained in a similar study with µ-selective opioid fentanyl in rats [206]. Moreover, JMV2959 pretreatment reduced the morphine-induced accumbens dopamine release and hyperlocomotion in mice [207]. Furthermore, JMV2959 significantly attenuated subchronic morphine-induced dopaminergic sensitization in the NAC shell/core as well as behavioral sensitization in rats [208]. It was also reported that JMV2959 pretreatment significantly reduced the expression of morphine-conditioned place preference (CPP) in mice and rats [207,208] and significantly dose-dependently reduced the manifestation and development of fentanyl-CPP in rats [206].

Another opioid self-administration study used a fentanyl IVSA model in rats. JMV2959 pretreatement significantly attenuated ongoing fentanyl self-administration, and also reduced fentanyl-seeking/relapse-like behavior tested in rats tested on the 12th day of the forced abstinence period [206]. A recent study that used a modification of the rat CPP test indicated that GHS-R1A antagonism might effectively prevent the morphine-induced memory reconsolidation and the relapse-like behavior [209]. One day after the last drug conditioning, a high JMV2959 dose (6 mg/kg) was administered immediately after the rat re-exposure to the morphine-paired chamber (drug-memory reactivation) and the CPP was retested 1 day and 7 days after the memory reactivation. The significant reduction in the CPP expression within this JMV2959-injected rat group indicated that ghrelin antagonism could prevent/inhibit the drug seeking after the drug withdrawal.

Engel et al. [207], investigated the impact of subchronic intraperitoneal treatment of acyl-ghrelin or GHS-R1A antagonist (JMV2959) on opioid peptide levels, specifically, Met-enkephaline Arg6Phe7 (MEAP), Leu-enkephalin-Arg6 (LeuArg), dynorphin B (DynB), in reward and stress-related brain regions. In comparison to saline, five days of systemic ghrelin administration did not significantly alter any of these peptide levels. Interestingly, five days of JMV2959 administration increased the δ-receptor active peptide levels in the brain reward areas VTA (namely, the MEAP), and striatum (the LeuArg), and the κ-receptor active peptide was increased in the hippocampus (DynB), with no differences in the amygdala and prefrontal cortex [207]. The authors hypothesized, that JMV2959 prevented the morphine-induced reward via stimulation of δ-receptor active peptides in striatum and VTA, and that the hippocampal κ-receptor-activating peptides might be involved in JMV2959’s ability to regulate memory formation of reward. The µ-receptor-activating peptides were not explored at this study. Further research is essential to elucidate the exact ghrelin—opioid relationships.

Other microdialysis rat experiments have investigated the potential role of ghrelin in modulating the cross-talk between opioids and endocannabinoid or GABAergic pathways [210]. The results documented that pretreatment with a GHS-R1A antagonist (JMV2959 i.p.) reversed both the acute and subchronic systemic morphine-induced increases in anandamide (AEA) levels in the NAC, intensified the acute morphine-induced decreases in 2-arachidonoyl glycerol (2-AG) levels, but attenuated the subchronic morphine-induced accumbens 2-AG decreases [210]. In the latter study with acute fentanyl administration, JMV2959 was microinjected into the VTA or infused into the NAC shell [211]. These experiments showed that JMV2959 pretreatment with the μ-selective opioid fentanyl mainly confirmed the previous results obtained from experiments using morphine, with the JMV2959 effects more expressed when administered into the NAC shell in comparison to the VTA. JMV2959 pretreatment also significantly decreased the fentanyl-evoked accumbens GABA efflux and reduced concurrently monitored fentanyl-induced behavioral stimulation. These results encourage further investigation to assess if substances affecting GABA or endocannabinoid concentrations and action, such as GHS-R1A antagonists, could be used to prevent opioid-seeking behavior [211].

### 4.2. Clinical Studies

Thus far, only one clinical study investigated the plasma acyl- and des-acyl ghrelin levels in opioid-dependent male adult patients (in the Turkish population). Ultimately, only 28 patients finished the study (23 patients voluntarily left the research group), and all were treated with buprenorphine together with naloxone. Both the patients and the matching healthy controls were tobacco smokers. Throughout the study, fasting blood samples were collected in total three times: on the first day of treatment, on the seventh day (at the end of the detox), and on the twenty-first day. No significant differences in the plasma acylated and unacylated-ghrelin levels between the opioid addicts and the healthy controls were found. Furthermore, no significant relationships between opioid craving and ghrelin were observed. At this time, it is extremely difficult to create a sufficiently large group solely compromised of opioid users who would be able to complete an entire study; however, further research is necessary to extend these rather preliminary data and obtain sufficiently complex picture (e.g., with the involvement of more patients, including women, and the exclusion of potentially interfering medication).

### 4.3. Summary

The relevant reviewed preclinical and clinical results are summarized in Table 3. (See also Tables S1 and S2 in the Supplementary Information). In general, recent preclinical studies suggest that ghrelin/GHS-R1As play an important role in the reinforcing effects of opioids/µ-agonists and imply the involvement of rather complex mesolimbic mechanisms, with the participation of several neurotransmitter systems (e.g., ghrelin, endocannabinoid, GABA, endogenous opioid systems). In addiction models, the GHS-R1A antagonism reduced the opioid (morphine and fentanyl) reward as well as the signs of craving/desire for opioids, and significantly attenuated the opioid (fentanyl and heroin) intake and drug-seeking behavior and prevented opioid-induced memory reconsolidation and relapse-like behavior (morphine). Further research, particularly in humans, is essential to explore the exact nature and mechanisms of ghrelin/GHS-R1A involvement in opioid addiction processes and the possible future use of ghrelin antagonism in the prevention and treatment of opioid addiction.

## 5. Ghrelin/GHS-R1As and Cannabinoids

The use of recreational and medical cannabis, together with social, medical, and legal acceptance of cannabis, have grown dramatically during the past 15 years in Europe and North America, despite the apparent consequent risks, including addiction [147,220]. Abused cannabinoids include, beside the natural constituents of cannabis (Cannabis sativa), a number of synthetic cannabinoids used in different ways, e.g., in herbal mixtures, infused papers, or even as adulterating cannabis with synthetic cannabinoids. Surveys from the general population in European Union revealed that around 1 % of adults were (almost) daily cannabis users, and 60 % of these users were under 35 years of age. Recently, it was estimated that about 9% of chronic cannabis users display characteristic symptoms of dependence [221]. Since 2014, various synthetic cannabinoids have stably represented roughly one third of all EMCDDA-monitored synthetic drugs abused in Europe, and many of them are associated with serious overdoses or even deaths [3,147]. Treatment of cannabinoid use disorders remains only symptomatic and unsatisfactory [222].

Cannabinoids mediate their effects by stimulating the complex endocannabinoid system, which consists of several binding sites (e.g., cannabinoid receptors CB1, CB2), a series of endogenous ligands/endocannabinoids (e.g., anandamide/AEA, 2-AG), and central and peripheral effects. Beside other roles, endocannabinoid system participates significantly in metabolism and homeostatic as well as hedonic food intake and drug abuse [22,221,223,224]. Ghrelin/GHS-R1As, and cannabinoid CB1R in particular, are distributed within the overlapping brain regions that are crucial for feeding (hypothalamus), reward, and motivation (VTA, NAC), and it has already been documented that both systems mutually interact to a significant extent in the regulation of homeostatic as well as hedonic food intake [200,225,226,227]. A clear interaction between ghrelin and cannabinoid mesolimbic signaling was documented by a study wherein systemic pretreatment with the CB1R antagonist/rimonabant significantly reduced intracerebroventricular ghrelin-induced NAC dopamine release and hyperlocomotion in mice [228].

Several recent studies have described the role of ghrelin/GHS-R1A in the cannabinoid addiction experimental rat models. Few studies have explored the effect of cannabis use on ghrelin blood levels in humans.

### 5.1. Preclinical Studies

Initial studies showed that a single intragastric administration of alcohol extract of Cannabis sativa [213], as well as a single intraperitoneal injection of the synthetic cannabinoid CB1 receptor agonists methanandamide and CP55940 [214], increased total ghrelin blood concentrations and food intake in rats.

A recent complex study [215] tested the effects of acyl-ghrelin and the ghrelin antagonism in several cannabinoid addiction rat models. It was found that pretreatment with the GHS-R1A antagonist JMV2959 significantly and dose-dependently reduced the manifestation of Δ-9-tetrahydrocannabinol/THC-conditioned place preference (CPP). THC-CPP development was also reduced after the simultaneous administration of JMV2959 and THC during conditioning. JMV2959 also significantly reduced THC-induced behavioral stimulation in the LABORAS cage [215], similarly to a later experiment involving the synthetic aminoalkylindol cannabinoid WIN55,212-2-induced behavioral stimulation [216]. Furthermore, JMV2959 pretreatment significantly attenuated the WIN55,212-2 intravenous self-administration (IVSA) in rats [215]. Pretreatment with JMV2959 also seemed to reduce WIN55,212-2-seeking/relapse-like behavior in rats tested on the 12th day of the forced abstinence period. On the contrary, pretreatment with systemic acyl-ghrelin significantly increased the cannabinoid IVSA as well as enhanced the relapse-like behavior. Co-administration of acyl-ghrelin and JMV2959 abolished/reduced any significant efficacy of the GHS-R1A antagonist in cannabinoid IVSA [215], which confirmed the involvement of GHS-R1As in the observed effects.

Recent microdialysis study in rats [216] found that the administration of WIN55,212-2 into the posterior VTA induced significant dopamine release in the NAC shell, which was significantly reduced by systemic JMV2959 pretreatment. Simultaneously, the cannabinoid-increased accumbens dopamine metabolic turnover was significantly augmented by JMV2959 pretreament, with the monoamine oxidase (MAO) metabolism products and their turnover being the most increased (DOPAC/dopamine, HVA/dopamine). Intracerebral WIN55,212-2 administration also increased the anandamide (AEA) and the 2-AG extracellular levels in the NAC shell, which were moderately but significantly attenuated by JMV2959 pretreatment. Moreover, the cannabinoid-induced decrease in accumbens-GABA levels was reversed by JMV2959 pretreatment [216].

### 5.2. Clinical Studies

Human studies have explored the impact of cannabinoids on ghrelin blood levels. Increased blood levels of total ghrelin were observed in chronic THC-smoking HIV-infected men [217]. Furthermore, increased blood levels of total ghrelin were observed after the oral administration of cannabis in healthy adult cannabis users [218]. Another study found that the total ghrelin blood concentrations after oral cannabis administration were higher than the smoked- and vaporized-cannabis-induced blood levels, while no significant effects on acyl-ghrelin were observed [219]. During the vaporized session, the THC area under curve (AUC) was positively correlated with the total ghrelin AUC and the positive correlation trend was apparent with the acyl-ghrelin AUC as well [219].

### 5.3. Summary

The relevant reviewed preclinical and clinical results have been summarized in Table 3. (See also Tables S1 and S2 in the Supplementary Information). The existing preclinical studies collectively suggest the importance of the involvement of ghrelin/GHS-R1As in the rewarding and reinforcing effects of cannabinoids/CB1-agonists. The studies imply the potential cooperation of ghrelin signaling with other neurotransmitter systems (e.g., the endocannabinoid, and GABA systems) within the NAC in the reinforcing effects of cannabinoids. The summarised experimental results also indicated that GHS-R1A antagonists deserve to be further elucidated as potential novel treatment strategies for cannabinoid addiction. The GHS-R1A antagonist JMV2959 significantly reduced several parameters of cannabinoid reward and attenuated cannabinoid intake and drug-seeking behavior. All currently available results regarding the investigation of acyl-ghrelin and its antagonist effects in cannabinoid addiction models originate from one research facility, nevertheless, they were obtained from a comprehensive dual investigation in several WIN55,212-2 and THC rat models [215,216]. Further preclinical and (particularly) clinical research is essential.

## 6. Discussion

In the present review, we aimed to provide a comprehensive overview of ghrelin /GHS-R1As’ possible involvement in the reinforcing effects of and addiction to nonalcohol addictive drugs. We did not focus on alcohol-ghrelin relationship, as the most advanced and extensive research into ghrelin-alcohol interactions was recently reviewed elsewhere [26,93,95], including the initial human study that tested one GHS-R1A inverse agonist in alcohol addicts; thus, we only mentioned the latest clinical results of that ongoing research [71,97,98].

Current drug-dependence pharmacotherapies, particularly relapse prevention, remain limited, unsatisfactory, unreliable for opioids and tobacco, and even symptomatic for stimulants and cannabinoids. Thus, ghrelin antagonism, a recently studied novel alcohol addiction treatment strategy, has been intensively explored in experimental models of various addictive drugs, including nicotine, stimulants, opioids, and cannabinoids (for references see Table 1, Table 2 and Table 3, and Table S1 in the Supplementary Information). The role of ghrelin signaling in the effects of these drugs has also been investigated. To our knowledge, research into the ghrelin system in the context of hypnotics, anxiolytics, inhalants, caffeine, or other addictive substances has not yet been published.

The investigation of the relationships between ghrelin and nonalcohol addictive drugs is still in its early stage, especially at the clinical level. Nevertheless, the majority of the reviewed experimental results obtained from rodents indicated the importance of the role of ghrelin signaling in the pro-addictive effects of nonalcohol abused drugs. Firstly, acyl-ghrelin (a GHS-R1A natural agonist) administration augmented the drug-induced reward/reinforcing effects in all the reviewed drug types (for references see Table 1, Table 2 and Table 3, and Table S1 in the Supplementary Information). Secondly, GHS-R1A antagonists attenuated the drug-induced reinforcement-associated effects in all the reviewed addictive drugs. The centrally administered peptide D-Lys3-GHRP6 was effective in the majority of the drug addiction models, and the triazole derivative JMV2959 [127] (administered systemically or centrally) was effective in all the described drug addiction models. Importantly, the usual effective JMV2959 doses (1–3 mg/kg i.p. in rats, 6 mg/kg in mice) did not induce significant neurobehavioral changes per se in the animal models. Future studies should use another GHS-R1A antagonist/inverse agonist to confirm the current results. The ghrelin antagonist decreased the drug-induced hyperlocomotion, the CPP, and the accumbens dopamine release in nicotine, stimulants, opioids, and cannabinoids. Furthermore, it reduced the self-administration (IVSA) of stimulants (methamphetamine and cocaine), opioids (fentanyl and heroin), and cannabinoids (WIN55,212-2) and attenuated the drug-seeking behavior in these IVSA models (for references see Table 1, Table 2 and Table 3, and Table S1 in the Supplementary Information). Studies using ghrelin and ghrelin antagonists in nicotine self-administration models are not yet available. Genetic manipulations (GHS-R1A and GHRL gene knockouts) reduced the marks of cocaine reinforcement [176,186]. Collectively, these results encourage further research, possibly aiming at a potential new ghrelin/GHS-R1A-related relapse-preventive treatment for these addictions.

Concerning the addictive drug’s impact on ghrelin blood levels, the majority of the experimental studies measured a more stable total ghrelin content. However, acyl-ghrelin is considered to be a GHS-R1A agonist [33]. Despite the suggested correlations between acyl- and des-acyl ghrelin [229], the acyl-ghrelin content should be the predominant focus in future studies, together with a more thorough standardized methodology, which might improve the consistency of the obtained results (see the suggestions within the human studies below). However, the total or acyl-ghrelin blood levels usually (but not always) increased following the nicotine/tobacco smoke, stimulant, or cannabinoid administration; the effects of opioids on ghrelin content have not yet been published (for references see Table 1, Table 2 and Table 3).

In terms of future experimental directions, arguments increasingly suggest that instead of measuring the ghrelin in the blood, the ghrelin content should be measured within the specific brain structures that express GHS-R1As [42], which are involved in the reward/reinforcement processes (e.g., the VTA, NAC, LDTg, amygdala) and are implicated in addiction pathophysiology (e.g., the hippocampus). The enhanced reinforcing effects of addictive drugs after the acyl-ghrelin administration into the VTA or NAC have been described (see Table 1, Table 2 and Table 3). However, as was also mentioned in this review, the evidence for the transfer of peripheral ghrelin into the deep brain reward structures (the VTA and NAC) [53] or synthesis of ghrelin within the brain in an effective amount [43] remains dubious. Therefore, such experiments in drug addiction models could significantly help to understand the specific mechanisms involved.

Existing human studies have searched for correlations between the ghrelin system and nonalcohol drug addiction (summarized in Table 1, Table 2 and Table 3, and Table S2 in Supplementary Information). Given the hereditary component underlying addictive disorders, several studies have explored the potential relationship between nicotine and stimulants-related outcomes and genetic variations of ghrelin signaling, through analyzing the frequency of single-nucleotide polymorphisms (SNPs) located in the pre-proghrelin gene (GHRL) and/or the GHSR-1A gene (GHSR). Ghrelin-related genetic studies in opioid and cannabinoid addictions are not yet available (see Table 3). Interestingly, correlations related to the GHSR-1A gene usually appear more convincing and robust compared to the ghrelin GHRL gen, which seems to be a consistent pattern across different drugs of abuse including nicotine, stimulants and also alcohol (see the references in Table 1 and Table 2 and elsewhere) [101,131]. However, as far as is currently known, the genetic variability of the ghrelin signaling system can hardly serve as a diagnostic marker for drug dependence or severity of drug use. Nonetheless, the present results strengthen the conception of ghrelin/GHS-R1A involvement in development of addictive behaviors, which might serve as suitable targets for new treatments of these disorders.

In addition, the majority of the clinical studies that explored the correlations between different phases of nicotine/tobacco and cannabis/THC use and ghrelin blood levels measured the total ghrelin content instead of the acyl-ghrelin, and the present results are rather variable (see Table 1, Table 2 and Table 3, and Table S2 in Supplementary Information). Previously, it was well documented that the ghrelin blood levels fluctuate throughout the day and can be affected by multiple circumstances [110], e.g., feeding state [38,39,156]; mood and stress factors, including stress history (e.g., early life adversity) [87,145]; age; gender; and hormonal changes [110]. Further factors should also be considered, such as the timing that had elapsed since the drug administration, the length of prior drug intake, the length of the abstinence period, the concurrent use of other drugs or medications, and comorbidities. The lack of controling for some of these factors could perhaps explain the variable results of the reviewed human studies (see Table 1, Table 2 and Table 3, and Table S2 in Supplementary Information). Some of the methodological variabilities or inconsistencies might have been caused by the different principal goals of the study (e.g., metabolic, endocrinologic, cardiovascular), or poor knowledge the significance of some factors at the time of the study’s origin (e.g., the importance of fasting blood sampling, or the influence of stress, which was only considered in one study [145]), or technical limits (e.g., acyl-ghrelin shows a lower stability and around a tenfold lower concentration in the blood in comparison to total ghrelin [229]). Future human studies should reflect as many as possible of the abovementioned factors, using more rigorous methodologies to gain more comparable results.

The present results, which were obtained using substances from diverse drug groups (i.e., substances of different effects and structures), together with previously published studies on the participation of ghrelin/GHS-R1As in food reinforcement [34,197], and cue-induced palatable food-intake reinstatement [230], and similar findings from alcohol-ghrelin/GHS-R1A studies [26,231], indicate that ghrelin and especially GHS-R1As seem to play an important role in the general reward, reinforcement, and craving mechanisms. However, further research is necessary.

Thus far, no human study has tested the possible ability of ghrelin antagonism to reduce consumption or craving behavior of nicotine, stimulants, opioids or cannabinoids (see Table 1, Table 2, Table 3, and Table S2 in Supplementary Information). Nonetheless, this should be expected, considering that the research is still in its early preclinical phase and that the only orally applicable ghrelin antagonist/inverse agonist that has demonstrated sufficient safety for use in clinical testing has only just been used in human studies for the first time [94,95,97,98]. First and foremost, further research is necessary to verify both the efficacy and safety of the prolonged/long-lasting use of ghrelin antagonism/inverse agonism in experimental as well as clinical conditions, which is currently lacking [26,95,101]. Even though the 14 days use of the GHS-R1A inverse agonist in alcohol-addicted patients did not require discontinuation due to adverse reactions [97], the complexity of central and peripheral ghrelin’s physiological roles [38] implies possible prospective unwanted consequences of (prolonged) GHS-R1A-blocking therapy. Metabotropic GHS-R1A signals through Gq, Gi/o, G13, and arrestin [67,232]. Relevant future research aiming at designing drugs that selectively target individual signaling pathways of the GHS-R1A might lead to the development of selective drugs to treat only the therapeutically relevant function (addiction) with minimal side effects [67]. Furthermore, the use of lower treatment doses of ghrelin antagonists/partial agonists should restrict the incidence and intensity of its possible adverse effects; thus, combinational drug development should be encouraged, e.g., inspired by the formation of oligomers and/or heterodimers of GHS-R1A with other metabotropic receptors [72,74]. Such specific receptor co-operations might achieve more targeted intensified effects through lowered doses and/or suitably influence some adverse addictive-drug-use consequences such as anxiety and memory disturbances [72]. Similarly, further investigation of the recently described modulatory relationships between ghrelin signaling and endogenous opioids [207], endocannabinoids, and GABA [211,216] (see Table 2 and Table 3) within drug reinforcement processes might also provide useful information or tools for the prevention of drug-seeking behaviors (including possible advantageous combinations). Furthermore, it was suggested, that inhibition of monoamine oxidase (MAO) by constituents contained in tobacco and tobacco smoke enhances the addiction induced by tobacco smoking [233]. Thus, the increase of MAO activity following ghrelin antagonist administration in the drug addiction models that were reviewed above [205,206,216] might contribute to some of the antiaddictive effectiveness of GHS-R1A antagonism (e.g., in tobacco addiction). At the same time, variable alteration of mitochondrial function was suggested in addictive disorders, resulting in epigenetic modifications and changes in gene expression, which are involved in addiction development [234]. MAO is localised on the outer mitochondrial membrane [235], and it was reported that the enzymatic metabolism of neurotransmitters by MAO can damage mitochondria and generate ROS [236,237]. Recently, ghrelin’s protective effects on mitochondrial functions have been indicated [238]. The effects of ghrelin antagonism on mitochondrial functions require further study, which should be encouraged from the perspectives of effectiveness and safety. Furthermore, the ghrelin-independent role of GHS-R1A’s constitutive activity in the (drug) reinforcement processes should be thoroughly studied, in consideration of the low ghrelin content within the reward brain structures [58]. Indeed, as was already mentioned, one ghrelin inverse agonist has recently been tested in human studies [71].

In conclusion, the reviewed results indicate that ghrelin and especially GHS-R1As seem to play an important role in the reinforcement and addiction behaviors of nicotine, stimulants, opioids, and cannabinoids. However, further preclinical and (mainly) clinical research is necessary to elucidate the exact mechanisms involved and to verify effectiveness and safety of the potential future utilization of GHS-R1A antagonism for these drug addiction therapies, particularly for reducing the risk of relapse.

## Figures and Tables

**Table 1:** Ghrelin/GHS-R1As and nicotine/tobacco—preclinical and clinical studies overview.

**PRECLINICAL STUDIES—NICOTINE/TOBACCO**			
**Study Design**	**Results**	**Drug of Abuse**	**Reference**
**Drug effect on the ghrelin blood concentrations**	Four-weeks (5 days a week) cigarettes smoke exposure increased acyl-ghrelin blood levels, while des-acyl ghrelin remained unaffected (in rats)	Nicotine/tobacco smoke	Tomoda et al. [123]
	Total ghrelin blood levels were not affected by the four-weeks cigarettes smoke exposure (in rats)	Nicotine/tobacco smoke	Ypsilantis et al. [124]
	(Total) ghrelin serum levels were increased following 3-weeks of nicotine administration (oral, inhaled, intraperitoneal) (in rats)	Nicotine	Ali et al. [125]
**Acyl-ghrelin administration effects**	Ghrelin amplified the nicotine-induced dopamine release in striatum slices (in vitro rat brain slices)	Nicotine	Palotai et al. [85]
**GHS-R1A antagonist administration**	Systemic JMV2959 pretreatment decreased the drug-induced hyperlocomotion, conditioned place preference expression (CPP) and accumbens dopamine release (in mice)	Nicotine	Jerlhag and Engel [128]
	Systemic JMV2959 pretreatment decreased the drug-induced behavioral sensitization	Nicotine	Wellman et al. [129]
**CLINICAL STUDIES—NICOTINE/TOBACCO**			
**Study Design**	**Results**	**Drug of Abuse**	**Reference**
**Genetic study**	Two haplotypes of the GHSR gene were associated with smoking status (Swedish population)	Nicotine/tobacco	Landgren at al. [130]
**Ghrelin blood levels**	Chewing nicotine gum in healthy non-smokers did not affect the blood total/des-acyl ghrelin levels	Nicotine	Kroemer et al. [121] Pilhatsch et al. [135]
	Acute cigarette smoking did not affect total ghrelin levels in non-smokers, but ghrelin was significantly reduced in saliva	Nicotine/tobacco	Kaabi and Khalifa [134]
	Acute cigarettes smoking decreased total ghrelin levels in non-smokers (no effect in habitual smokers)	Nicotine/tobacco	Kokkinos et al. [133]
	Acute cigarette smoking increased total ghrelin levels in smokers and non-smokers	Nicotine/tobacco	Bouros et al. [132]
	Higher fasting total ghrelin serum levels in active smokers than in former and never-smokers (German population)	Nicotine/tobacco	Wittekind et al. [141]
	No correlation was found between total ghrelin and tobacco smoking (habituated smokers) (Finish population//Poykko et al.)	Nicotine/tobacco	Bouhours-Nouet et al. [136] Kokkinos et al. [133] Poykko et al. [137]
	Concentration of acyl-ghrelin, but not total ghrelin, was significantly higher in habituated smokers than in non-smokers	Nicotine/tobacco	Koopmann et al. [138]
	Increased total ghrelin blood levels were observed in current smokers in comparison to non-smokers	Nicotine/tobacco	Wittekind et al. [141] Fagerberg et al. [139] Langenberg et al. [140] al’Absi et al. [145]
	Total ghrelin blood levels may predict the risk of smoking relapse	Nicotine/tobacco	al’Absi et al. [142] Lemieux and al’Absi [143]
	Decreases in acyl-ghrelin levels during abstinence from tobacco in comparison to levels prior to smoking cessation	Nicotine/tobacco	Lee et al. [144]
	In comparison to non-smokers, active smoking seemed to be positively associated with total ghrelin levels, which were attenuated during smoking withdrawal	Nicotine/tobacco	al’Absi et al. [145]

**Table 2:** Ghrelin/GHS-R1As and stimulants—preclinical and clinical studies overview.

**PRECLINICAL STUDIES—STIMULANTS**			
**Study Design**	**Results**	**Drug of Abuse**	**Reference**
**Drug effect on the ghrelin blood concentrations**	Total ghrelin blood levels were increased following drug single dose administration (in rats)	Methamphetamine	Crowley et al. [155]
		Methamphetamine and high doses of MDMA	Kobeissy et al. [156]
	Drug IVSA and extinction/anticipation significantly increased both acyl- and des-acyl ghrelin blood levels (in rats) Blockade of peripheral adrenergic ß1 receptors by atenolol attenuated the elevation in circulating ghrelin induced by cocaine	Cocaine	You et al. [158,159]
	Total ghrelin blood levels positively correlated with cue-induced cocaine-seeking behavior (in rats)	Cocaine (cue)	Tessari et al. [157]
**Acyl-ghrelin administration effects**	Systemic pretreatment with acute ghrelin augmented/sensitized acute cocaine-induced hyperlocomotion (in rats)	Cocaine	Wellman et al. [161]
	Systemic pretreatment with repeated ghrelin augmented/sensitized acute cocaine-induced hyperlocomotion (in rats)	Cocaine	Wellman et al. [162]
	Central (NAC-core) pretreatment with ghrelin augmented/sensitized acute drug-induced hyperlocomotion (in rats)	Cocaine	[167]
	Central (NAC-core) pretreatment with ghrelin augmented acute drug-induced hyperlocomotion and in co-administration with D1-agonist also the drug-induced behavioral senzitization (in rats)	Amphetamine	Jang et al. [168]
	Systemic pretreatment with ghrelin increased development of drug-induced place preference (CPP) (in rats)	Cocaine	Davis et al. [169]
	Central (VTA) pretreatment with ghrelin increased development of drug-induced place preference (CPP) (in rats)	Cocaine	Schuette et al. [170]
	Central (VTA and NAC) pretreatment with ghrelin increased development of drug-CPP and this was attenuated when JMV2959 was centrally co-administered with ghrelin (in rats)	Cocaine	Dunn et al. [171]
	Systemic and central (VTA) ghrelin augments cocaine-enhanced alcohol consumption (in rats)	Cocaine (alcohol)	Cepko et al. [172]
**GHS-R1A antagonist administration**	Systemic JMV2959 pretreatment decreased the drug-induced hyperlocomotion (in mice)	Cocaine and Amphetamine	Jerlhag et al. [175]
	Systemic JMV2959 pretreatment decreases the expression of drug-induced conditioned place preference expression (CPP) and accumbens dopamine release (in mice)	Cocaine and Amphetamine	Jerlhag et al. [175]
	Repeated systemic JMV2959 pretreatment decreases the drug-induced hyperlocomotion (in mice)	Amphetamine	Suchankova et al. [104]
	Systemic JMV2959 reduced systemic drug-induced increase of dopamine in the NAC-shell and in the VTA (in rats)	Amphetamine	Edvardsson et al. [178]
	Systemic repeated JMV2959 together with cocaine decreased the drug-induced behavioral sensitization (in rats)	Cocaine	Clifford et al. [176]
	Systemic JMV2959 pretreatment decreased the drug intravenous self-administration (IVSA) and drug-seeking behavior, plus the expression and also development of drug-induced conditioned place preference (CPP) (in rats)	Methamphetamine	Havlickova et al. [177]
	Systemic JMV2959 pretreatment decreased the drug-induced CPP, as well as the body weight gain. However, acyl-ghrelin antibodies administration attenuated the weight gain but not the cocaine-CPP, which indicated, that the GHS-R1A effects on reward are independent from peripheral acyl-ghrelin binding (in mice)	Cocaine	Wenthur et al. [54]
	Systemic JMV2959 pretreatment dose-dependently inhibited drug-IVSA, drug-seeking, and reinstatement of drug-seeking triggered by the drug (in rats)	Cocaine	You et al. [159]
	Systemic JMV2959 pretreatment inhibited the brain stimulation reward (BSR) and drug-potentiated BSR maintained by optogenetic stimulation of VTA dopamine (in mice)	Cocaine	You et al. [159]
**GHS-R1A knockout animals**	GHS-R1A gene knockouts show reduced drug-induced behavioral sensitization (rats)	Cocaine	Clifford et al. [176]
**Ghrelin peptide gene (GHRL) knockout animals**	GHRL gene knockouts showed reduced drug-induced hyperlocomotion as well as reduction of behavioral sensitization dopamine content in striatal dissections (30 min after cocaine) did not differ between GHRL knockouts and wild mice	Cocaine	Abizaid et al. [186]
**CLINICAL STUDIES—STIMULANTS**			
**Study Design**	**Results**	**Drug of Abuse**	**Reference**
**Genetic study**	No association between pre-proghrelin gene (GHRL) variations and the drug dependence, but correlation between SNP on GHRL and emotional problems (depression, anxiety) was found (Korean population)	Methamphetamine	Yoon et al. [182]
	SNP located on the ghrelin receptor gene GHSR seemed associated with the drug dependence; no differences were found between drug-dependent and healthy participants in SNP on the pre-proghrelin gene (GHRL) (Swedish population)	Amphetamine	Suchankova et al. [183]
**Ghrelin blood levels**	In children with ADHD the total ghrelin blood levels were increased after two months treatment with the drug in comparison to basal pretreatment levels	Methylphenidate	Sahin et al. [185]
	No significant effect of intravenous drug administration in experienced cocaine users on the acyl- or total ghrelin blood levels was observed	Cocaine	Bouhlal et al. [184]

**Table 3:** Ghrelin/GHS-R1As and opioids plus Ghrelin/GHS-R1As and cannabinoids—preclinical and clinical studies overview.

**PRECLINICAL STUDIES—OPIOIDS**			
**Study Design**	**Results**	**Drug of Abuse**	**Reference**
**Acyl-ghrelin administration effects**	Central (intracerebroventricular) pretreatment with acyl-ghrelin increased drug intravenous self-administration (IVSA) and drug-seeking (in rats)	Heroin	Maric et al. [203]
**GHS-R1A antagonist administration**	Systemic JMV2959 reduced systemic drug-induced increase of dopamine in the NAC-shell and hyperlocomotion, also it increased accumbens dopamine metabolism by MAO (in rats)	Morphine	Sustkova-Fiserova et al. [205]
		Fentanyl	Sustkova-Fiserova et al. [206]
	Systemic JMV2959 pretreatment decreased the expression of drug-induced conditioned place preference expression (CPP), hyperlocomotion and accumbens dopamine release (in mice)	Morphine	Engel et al. [207]
	Systemic JMV2959 reversed both the acute and sub-chronic systemic drug-induced increases in anandamide/AEA levels in the NAC, intensified acute drug-induced decreases in 2-AG levels but attenuated the sub-chronic drug-induced accumbens 2-AG decreases	Morphine	Sustkova-Fiserova et al. [210]
	Acute systemic JMV2959 and also central JMV2959 (VTA and NAC-shell) reversed the drug-induced anandamide/AEA increases in the NAC shell, and intensified drug-induced decreases in the NAC shell 2-AG levels, with both JMV2959 effects more expressed when administered into the NAC shell in comparison to the VTA. JMV2959 pre-treatment also decreased the drug-evoked accumbens GABA efflux (in rats)	Fentanyl	Sustkova-Fiserova et al. [211]
	Systemic JMV2959 reduces systemic subchronic drug-induced accumbens dopaminergic sensitization and behavioral sensitization and also drug-induced conditioned place preference (CPP) expression (in rats)	Morphine	Jerabek et al. [208]
	Systemic JMV2959 prevented the drug-induced memory reconsolidation and relapse-like behavior in the modified CPP method (in rats)	Morphine	Zhao et al. [209]
	Systemic JMV2959 pretreatment decreases the drug intravenous self-administration (IVSA) and drug-seeking behavior, plus the expression and also development of drug-induced conditioned place preference (CPP) (in rats)	Fentanyl	Sustkova-Fiserova et al. [206]
	Central (intracerebroventricular) D-Lys3-GHRP6 pretreatment had no effect on drug intravenous selfadministration (IVSA) or food deprivation-induced reinstatement of drug seeking	Heroin	Maric et al. [203]
	Central (into VTA) JMV2959 pretreatment decreased the drug-seeking in food restricted rats	Heroin	D’Cunha et al. [204]
**CLINICAL STUDIES—OPIOIDS**			
**Study Design**	**Results**	**Drug of Abuse**	**Reference**
**Ghrelin blood levels**	No significant differences in plasma acyl- and des-acyl ghrelin blood levels between opioid problematic users and healthy controls was found as well as no associations with opioid craving (Turkish population)	Heroin	Kara [212]
**PRECLINICAL STUDIES—CANNABINOIDS**			
**Study Design**	**Results**	**Drug of Abuse**	**Reference**
**Drug effect on the ghrelin blood concentration**	Intra-gastric drug administration increased total ghrelin blood levels (in rats)	Cannabis extract	Mazidi et al. [213]
	Systemic drug administration increased total ghrelin blood levels (in rats)	Synthetic cannabinoids Methanandamide and CP55940	Zbucki et al. [214]
**Acyl-ghrelin administration effects**	Systemic acyl-ghrelin increased the drug intravenous self-administration and drug-seeking behavior	Synthetic cannabinoid WIN55,212-2	Charalambous et al. [215]
**GHS-R1A antagonist administration**	Systemic JMV2959 pretreatment decreased the systemic drug-induced hyperlocomotion in rats	THC, synthetic cannabinoid WIN55,212-2	WIN55,212-2-Charalambous et al. [216] THC—Charalambous et al. [215]
	Systemic JMV2959 pretreatment decreased the drug intravenous self-administration (IVSA) and drug-seeking behavior, plus the expression and also development of drug-induced conditioned place preference (CPP) (in rats)	THC for CPP and WIN55,212-2 for IVSA	Charalambous et al. [215]
	Systemic JMV2959 pretreatment decreased the central (into VTA) drug-induced dopamine increase, anandamide/AEA and 2-AG increase and reversed the drug-induced GABA decrease in the NAC shell	synthetic cannabinoid WIN55,212,2	Charalambous et al. [216]
**CLINICAL STUDIES—CANNABINOIDS**			
**Study Design**	**Results**	**Drug of Abuse**	**Reference**
**Ghrelin blood levels**	Total ghrelin blood levels were increased in chronic drug smoking HIV patients	Chronic THC smokers	Riggs et al. [217]
	Total ghrelin blood levels were increased after oral drug administration in healthy cannabis users	Cannabis	Farokhnia et al. [218]
	Total ghrelin blood levels were higher after oral drug administration in comparison to smoked and vaporized drug; no significant effects on acyl-ghrelin were found	Cannabis	Farokhnia et al. [219]
	Vaporized drug AUC was positively correlated with total ghrelin AUC and a similar positive correlation between drug AUC and acyl-ghrelin AUC was also indicated	THC	Farokhnia et al. [219]

## Data Availability

Not applicable.

## Supplementary Materials

The following supporting information can be downloaded at: https://www.mdpi.com/article/10.3390/ijms23020761/s1.
