# Supplementary material for: The Role of Ghrelin/GHS-R1A Signaling in Nonalcohol Drug Addictions
Source: Int J Mol Sci. 2022 Jan 11;23(2):761. doi: 10.3390/ijms23020761 (PMC8776007; doi:10.3390/ijms23020761)
Supplement: Supplementary file 1 [file ijms-23-00761-s001.zip › ijms-1515840-supplementary.pdf]

***Supplementary Information** – The role of the ghrelin/GHS-R1A signaling in non-alcohol drug addictions (REVIEW) - M. Sustkova-Fiserova et al.*

Table S1. PRECLINICAL STUDIES OVERVIEW

Study design	Drug	Results	Drug of abuse	Reference
**Drug effect on the ghrelin blood concentrations**	**N**	Four-weeks (5 days a week) cigarettes smoke exposure increased acyl-ghrelin blood levels, while desacyl ghrelin remained unaffected (in rats)	Nicotine/tobacco smoke	Tomoda et al. [1]
	**N**	Total ghrelin blood levels were not affected by the four-weeks cigarettes smoke exposure (in rats)	Nicotine/tobacco smoke	Ypsilantis et al. [2]
	**N**	(Total) ghrelin serum levels were increased following 3-weeks of nicotine administration (oral, inhaled, intraperitoneal) (in rats)	Nicotine	Ali et al. [3]
	**S**	Total ghrelin blood levels were increased following drug single dose administration (in rats)	Methamphetamine	Crowley et al. [4]
	**S**		Methamphetamine and high doses of MDMA	Kobeissy et al. [5]
	**S**	Drug IVSA and extinction/anticipation significantly increased both acyl- and desacyl ghrelin blood levels (in rats)<br>Blockade of peripheral adrenergic ß1 receptors by atenolol attenuated the elevation in circulating ghrelin induced by cocaine	Cocaine	You et al. [6, 7]
	**S**	Total ghrelin blood levels positively correlated with cue-induced cocaine-seeking behavior (in rats)	Cocaine (cue)	Tessari et al. [8]
	**C**	Intra-gastric drug administration increased total ghrelin blood levels (in rats)	Cannabis extract	Mazidi et al. [9]
	**C**	Systemic drug administration increased total ghrelin blood levels (in rats)	Synthetic cannabinoids Methanandamide and CP55,940	Zbucki et al. [10]
**Acyl-ghrelin administration effects**	**N**	Ghrelin amplified the nicotine-induced dopamine release in striatum slices (in vitro rat brain slices)	Nicotine	Palotai et al. [11]
	**S**	Systemic pretreatment with acute ghrelin augmented/sensitized acute cocaine-induced hyperlocomotion (in rats)	Cocaine	Wellman et al. [12]
	**S**	Systemic pretreatment with repeated ghrelin augmented/sensitized acute cocaine-induced hyperlocomotion (in rats)	Cocaine	Wellman et al. [13]
	**S**	Central (NAC-core) pretreatment with ghrelin augmented/sensitized acute drug-induced hyperlocomotion (in rats)	Cocaine	Jang et al. [14]
	**S**	Central (NAC-core) pretreatment with ghrelin augmented acute drug-induced hyperlocomotion and in co-administration with D1-agonist also the drug-induced behavioral senzitization (in rats)	Amphetamine	Jang et al. [15]
	**S**	Systemic pretreatment with ghrelin increased development of drug-induced place preference (CPP) (in rats)	Cocaine	Davis et al. [16]
	**S**	Central (VTA) pretreatment with ghrelin increased development of drug-induced place preference (CPP) (in rats)	Cocaine	Schuette et al. [17]
	**S**	Central (VTA and NAC) pretreatment with ghrelin increased development of drug-CPP and this was attenuated when JMV2959 was centrally co-administered with ghrelin (in rats)	Cocaine	Dunn et al. [18]

	**S**	Systemic and central (VTA) ghrelin augments cocaine-enhanced alcohol consumption (in rats)	Cocaine (alcohol)	Cepko et al. [19]
	**O**	Central (intracerebroventricular) pretreatment with acyl-ghrelin increased drug intravenous self-administration (IVSA) and drug-seeking (in rats)	Heroin	Maric et al. [20]
	**C**	Systemic acyl-ghrelin increased the drug intravenous self-administration and drug-seeking behavior	Synthetic cannabinoid WIN55,212-2	Charalambous et al. [21]
**GHS-R1A antagonist administration**	**N**	Systemic JMV2959 pretreatment decreased the drug-induced hyperlocomotion, conditioned place preference expression (CPP) and accumbens dopamine release (in mice)	Nicotine	Jerlhag and Engel [22]
	**N**	Systemic JMV2959 pretreatment decreased the drug-induced behavioral sensitization	Nicotine	Wellman et al. [23]
	**S**	Systemic JMV2959 pretreatment decreased the drug-induced hyperlocomotion (in mice)	Cocaine and Amphetamine	Jerlhag et al. [24]
	**S**	Systemic JMV2959 pretreatment decreases the expression of drug-induced conditioned place preference expression (CPP) and accumbens dopamine release (in mice)	Cocaine and Amphetamine	Jerlhag et al. [24]
	**S**	Repeated systemic JMV2959 pretreatment decreases the drug-induced hyperlocomotion (in mice)	Amphetamine	Suchankova et al. [25]
	**S**	Systemic JMV2959 reduced systemic drug-induced increase of dopamine in the NAC-shell and in the VTA (in rats)	Amphetamine	Edvardsson et al. [26]
	**S**	Systemic repeated JMV2959 together with cocaine decreased the drug-induced behavioral sensitization (in rats)	Cocaine	Clifford et al. [27]
	**S**	Systemic JMV2959 pretreatment decreased the drug intravenous self-administration (IVSA) and drug-seeking behavior, plus the expression and also development of drug-induced conditioned place preference (CPP) (in rats)	Methamphetamine	Havlickova et al. [28]
	**S**	Systemic JMV2959 pretreatment decreased the drug-induced CPP, as well as the body weight gain. However, acyl-ghrelin antibodies administration attenuated the weight gain but not the cocaine-CPP, which indicated, that the GHS-R1A effects on reward are independent from peripheral acyl-ghrelin binding. (in mice)	Cocaine	Wenthur et al. [29]
	**S**	Systemic JMV2959 pretreatment dose-dependently inhibited drug-IVSA, drug-seeking, and reinstatement of drug-seeking triggered by the drug (in rats)	Cocaine	You et al. [6]
	**S**	Systemic JMV2959 pretreatment inhibited the brain stimulation reward (BSR) and drug-potentiated BSR maintained by optogenetic stimulation of VTA dopamine (in mice)	Cocaine	You et al. [6]
	**O**	Systemic JMV2959 reduced systemic drug-induced increase of dopamine in the NAC-shell and hyperlocomotion, also it increased accumbens dopamine metabolism by MAO (in rats)	Morphine	Sustkova-Fiserova et al. [30]
	**O**		Fentanyl	Sustkova-Fiserova et al. [31]
	**O**	Systemic JMV2959 pretreatment decreased the expression of drug-induced conditioned place preference expression (CPP), hyperlocomotion and accumbens dopamine release (in mice)	Morphine	Engel et al. [32]
	**O**	Systemic JMV2959 reversed both the acute and sub-	Morphine	Sustkova-

		chronic systemic drug-induced increases in anandamide/AEA levels in the NAC, intensified acute drug-induced decreases in 2-AG levels but attenuated the sub-chronic drug-induced accumbens 2-AG decreases		Fiserova et al. [33]
	**O**	Acute systemic JMV2959 and also central JMV2959 (VTA and NAC-shell) reversed the drug-induced anandamide/AEA increases in the NAC shell, and intensified drug-induced decreases in the NAC shell 2-AG levels, with both JMV2959 effects more expressed when administered into the NAC shell in comparison to the VTA.<br>JMV2959 pre-treatment also decreased the drug-evoked accumbens GABA efflux (in rats)	Fentanyl	Sustkova-Fiserova et al. [34]
	**O**	Systemic JMV2959 reduces systemic subchronic drug-induced accumbens dopaminergic sensitization and behavioral sensitization<br>and also drug-induced conditioned place preference (CPP) expression (in rats)	Morphine	Jerabek et al. [35]
	**O**	Systemic JMV2959 prevented the drug-induced memory reconsolidation and relapse-like behavior in the modified CPP method (in rats)	Morphine	Zhao et al. [36]
	**O**	Systemic JMV2959 pretreatment decreases the drug intravenous self-administration (IVSA) and drug-seeking behavior, plus the expression and also development of drug-induced conditioned place preference (CPP) (in rats)	Fentanyl	Sustkova-Fiserova et al. [31]
	**O**	Central (intracerebroventricular) D-Lys3-GHRP6 pretreatment had no effect on drug intravenous selfadministration (IVSA) or food deprivation-induced reinstatement of drug seeking	Heroin	Maric et al. [20]
	**O**	Central (into VTA) JMV2959 pretreatment decreased the drug-seeking in food restricted rats	Heroin	D´Cunha et al. [37]
	**C**	Systemic JMV2959 pretreatment decreased the systemic drug-induced hyperlocomotion in rats	THC, synthetic cannabinoid WIN55,212-2	WIN55,212-2 - Charalambous et al. [38]<br>THC - Charalambous et al. [21]
	**C**	Systemic JMV2959 pretreatment decreased the drug intravenous self-administration (IVSA) and drug-seeking behavior, plus the expression and also development of drug-induced conditioned place preference (CPP) (in rats)	THC for CPP and WIN55,212-2 for IVSA	Charalambous et al. [21]
	**C**	Systemic JMV2959 pretreatment decreased the central (into VTA) drug-induced dopamine increase, anandamide/AEA and 2-AG increase and reversed the drug-induced GABA decrease in the NAC shell	synthetic cannabinoid WIN55212,2	Charalambous et al. [38]
**GHS-R1A knockout animals**	**S**	GHS-R1A gene knockouts show reduced drug-induced behavioral sensitization (rats)	Cocaine	Clifford et al. [27]
**Ghrelin peptide gene (GHRL) knockout animals**	**S**	GHRL gene knockouts showed reduced drug-induced hyperlocomotion as well as reduction of behavioral sensitization<br>dopamine content in striatal dissections (30 min after cocaine) did not differ between GHRL knockouts and wild mice	Cocaine	Abizaid et al. [39]

**Table S2. CLINICAL STUDIES OVERVIEW**				
**Study design**	**Drug**	**Results**	**Substance**	**Reference**
**Genetic study**	**N**	Two haplotypes of the GHSR gene were associated with smoking status (Swedish population)	Nicotine/tobacco	Landgren et al. [40]
	**N**	Minor allele of the SNP located on the GHSR-1A gene (GHSR) but not on the pre-proghrelin gene (GHRL) appeared to be associated with increased severity of cigarette smoking (Swedish population)	Nicotine/tobacco	Suchankova et al. [41]
	**S**	No association between pre-proghrelin gene (GHRL) variations and the drug dependence, but correlation between SNP on GHRL and emotional problems (depression, anxiety) was found (Korean population)	Methamphetamine	Yoon et al. [42]
	**S**	SNP located on the ghrelin receptor gene GHSR seemed associated with the drug dependence; no differences were found between drug-dependent and healthy participants in SNP on the pre-proghrelin gene (GHRL) (Swedish population)	Amphetamine	Suchankova et al. [43]
**Ghrelin blood levels**	**N**	Chewing nicotine gum in healthy non-smokers did not affect the blood total/desacyl ghrelin levels	Nicotine	Kroemer et al. [44] Pilhatsch et al. [45]
	**N**	Acute cigarette smoking did not affect total ghrelin levels in non-smokers, but ghrelin was significantly reduced in saliva	Nicotine/tobacco	Kaabi and Khalifa [46]
	**N**	Acute cigarettes smoking decreased total ghrelin levels in non-smokers (no effect in habitual smokers)	Nicotine/tobacco	Kokkinos et al. [47]
	**N**	Acute cigarette smoking increased total ghrelin levels in smokers and non-smokers	Nicotine/tobacco	Bouros et al. [48]
	**N**	Higher fasting total ghrelin serum levels in active smokers than in former and never-smokers (German population)	Nicotine/tobacco	Wittekind et al. [49]
	**N**	No correlation was found between total ghrelin and tobacco smoking (habituated smokers) (Finish population//Poykko et al)	Nicotine/tobacco	Bouhours-Nouet et al. [50] Kokkinos et al. [47] Poykko et al. [51]
	**N**	Concentration of acyl-ghrelin, but not total ghrelin, was significantly higher in habituated smokers than in non-smokers	Nicotine/tobacco	Koopmann et al. [52]
	**N**	Increased total ghrelin blood levels were observed in current smokers in comparison to non-smokers	Nicotine/tobacco	Wittekind et al. [49] Fagerberg et al. [53] Langenberg et al. [54] al´Absi et al. [55]
	**N**	Total ghrelin blood levels may predict the risk of smoking relapse	Nicotine/tobacco	al´Absi et al. [56] Lemieux and al´Absi [57]
	**N**	Decreases in acyl-ghrelin levels during abstinence from	Nicotine/tob	Lee et al.

		tobacco in comparison to levels prior to smoking cessation	acco	[58]
	**N**	In comparison to non-smokers, active smoking seemed to be positively associated with total ghrelin levels, which were attenuated during smoking withdrawal	Nicotine/tob acco	al´Absi et al. [55]
	**S**	In children with ADHD the total ghrelin blood levels were increased after two months treatment with the drug in comparison to basal pretreatment levels	Methylpheni date	Sahin et al. [59]
	**S**	No significant effect of intravenous drug administration in experienced cocaine users on the acyl- or total ghrelin blood levels was observed	Cocaine	Bouhlal et al. [60]
	**O**	No significant differences in plasma acyl- and desacyl ghrelin blood levels between opioid problematic users and healthy controls was found as well as no associations with opioid craving (Turkish population)	Heroin	Kara [61]
	**C**	Total ghrelin blood levels were increased in chronic drug smoking HIV patients	Chronic THC smokers	Riggs et al. [62]
	**C**	Total ghrelin blood levels were increased after oral drug administration in healthy cannabis users	Cannabis	Farokhnia et al. [63]
	**C**	Total ghrelin blood levels were higher after oral drug administration in comparison to smoked and vaporized drug; no significant effects on acyl-ghrelin were found	Cannabis	Farokhnia et al. [64]
	**C**	Vaporized drug-AUC positively correlated with total ghrelin-AUC and similar trend-positive correlation of drug-AUC with acyl ghrelin AUC was observed	THC	Farokhnia et al. [64]

References:

1. Tomoda, K.; Kubo, K.; Nishii, Y.; Yamamoto, Y.; Yoshikawa, M.; Kimura, H., Changes of ghrelin and leptin levels in plasma by cigarette smoke in rats. *J Toxicol Sci* **2012,** 37, (1), 131-8.
2. Ypsilantis, P.; Politou, M.; Anagnostopoulos, C.; Tsigalou, C.; Kambouromiti, G.; Kortsaris, A.; Simopoulos, C., Effects of cigarette smoke exposure and its cessation on body weight, food intake and circulating leptin, and ghrelin levels in the rat. *Nicotine Tob Res* **2013,** 15, (1), 206-12.
3. Ali, S. S.; Hamed, E. A.; Ayuob, N. N.; Shaker Ali, A.; Suliman, M. I., Effects of different routes of nicotine administration on gastric morphology and hormonal secretion in rats. *Exp Physiol* **2015,** 100, (8), 881-95.
4. Crowley, W. R.; Ramoz, G.; Keefe, K. A.; Torto, R.; Kalra, S. P.; Hanson, G. R., Differential effects of methamphetamine on expression of neuropeptide Y mRNA in hypothalamus and on serum leptin and ghrelin concentrations in ad libitum-fed and schedule-fed rats. *Neuroscience* **2005,** 132, (1), 167-73.
5. Kobeissy, F. H.; Jeung, J. A.; Warren, M. W.; Geier, J. E.; Gold, M. S., Changes in leptin, ghrelin, growth hormone and neuropeptide-Y after an acute model of MDMA and methamphetamine exposure in rats. *Addict Biol* **2008,** 13, (1), 15-25.
6. You, Z. B.; Galaj, E.; Alen, F.; Wang, B.; Bi, G. H.; Moore, A. R.; Buck, T.; Crissman, M.; Pari, S.; Xi, Z. X.; Leggio, L.; Wise, R. A.; Gardner, E. L., Involvement of the ghrelin system in the maintenance and reinstatement of cocaine-motivated behaviors: a role of adrenergic action at peripheral beta1 receptors. *Neuropsychopharmacology* **2021**.
7. You, Z. B.; Wang, B.; Gardner, E. L.; Wise, R. A., Cocaine and cocaine expectancy increase growth hormone, ghrelin, GLP-1, IGF-1, adiponectin, and corticosterone while decreasing leptin, insulin, GIP, and prolactin. *Pharmacol Biochem Behav* **2019,** 176, 53-56.

8. Tessari, M.; Catalano, A.; Pellitteri, M.; Di Francesco, C.; Marini, F.; Gerrard, P. A.; Heidbreder, C. A.; Melotto, S., Correlation between serum ghrelin levels and cocaine-seeking behaviour triggered by cocaine-associated conditioned stimuli in rats. *Addict Biol* **2007,** 12, (1), 22-9.
9. Mazidi, M.; Baghban Taraghdari, S.; Rezaee, P.; Kamgar, M.; Jomezadeh, M. R.; Akbarieh Hasani, O.; Soukhtanloo, M.; Hosseini, M.; Gholamnezhad, Z.; Rakhshandeh, H.; Norouzy, A.; Esmaily, H.; Patterson, M.; Nematy, M., The effect of hydroalcoholic extract of Cannabis Sativa on appetite hormone in rat. *J Complement Integr Med* **2014,** 11, (4), 253-7.
10. Zbucki, R. L.; Sawicki, B.; Hryniewicz, A.; Winnicka, M. M., Cannabinoids enhance gastric X/A-like cells activity. *Folia Histochem Cytobiol* **2008,** 46, (2), 219-24.
11. Palotai, M.; Bagosi, Z.; Jaszberenyi, M.; Csabafi, K.; Dochnal, R.; Manczinger, M.; Telegdy, G.; Szabo, G., Ghrelin amplifies the nicotine-induced dopamine release in the rat striatum. *Neurochemistry international* **2013,** 63, (4), 239-43.
12. Wellman, P. J.; Davis, K. W.; Nation, J. R., Augmentation of cocaine hyperactivity in rats by systemic ghrelin. *Regulatory peptides* **2005,** 125, (1-3), 151-4.
13. Wellman, P. J.; Hollas, C. N.; Elliott, A. E., Systemic ghrelin sensitizes cocaine-induced hyperlocomotion in rats. *Regulatory peptides* **2008,** 146, (1-3), 33-7.
14. Jang, J. K.; Kim, W. Y.; Cho, B. R.; Lee, J. W.; Kim, J. H., Microinjection of ghrelin in the nucleus accumbens core enhances locomotor activity induced by cocaine. *Behav Brain Res* **2013,** 248, 7-11.
15. Jang, J. K.; Kim, W. Y.; Cho, B. R.; Lee, J. W.; Kim, J. H., Locomotor sensitization is expressed by ghrelin and D1 dopamine receptor agonist in the nucleus accumbens core in amphetamine pre-exposed rat. *Addict Biol* **2018,** 23, (3), 849-856.
16. Davis, K. W.; Wellman, P. J.; Clifford, P. S., Augmented cocaine conditioned place preference in rats pretreated with systemic ghrelin. *Regulatory peptides* **2007,** 140, (3), 148-52.
17. Schuette, L. M.; Gray, C. C.; Currie, P. J., Microinjection of Ghrelin into the Ventral Tegmental Area Potentiates Cocaine-Induced Conditioned Place Preference. *Journal of behavioral and brain science* **2013,** 3, (8), 276-580.
18. Dunn, D. P.; Bastacky, J. M. R.; Gray, C. C.; Abtahi, S.; Currie, P. J., Role of mesolimbic ghrelin in the acquisition of cocaine reward. *Neuroscience letters* **2019,** 709, 134367.
19. Cepko, L. C.; Selva, J. A.; Merfeld, E. B.; Fimmel, A. I.; Goldberg, S. A.; Currie, P. J., Ghrelin alters the stimulatory effect of cocaine on ethanol intake following mesolimbic or systemic administration. *Neuropharmacology* **2014,** 85, 224-31.
20. Maric, T.; Sedki, F.; Ronfard, B.; Chafetz, D.; Shalev, U., A limited role for ghrelin in heroin self-administration and food deprivation-induced reinstatement of heroin seeking in rats. *Addict Biol* **2012,** 17, (3), 613-22.
21. Charalambous, C.; Havlickova, T.; Lapka, M.; Puskina, N.; Slamberova, R.; Kuchar, M.; Sustkova-Fiserova, M., Cannabinoid-Induced Conditioned Place Preference, Intravenous Self-Administration, and Behavioral Stimulation Influenced by Ghrelin Receptor Antagonism in Rats. *International journal of molecular sciences* **2021,** 22, (5).
22. Jerlhag, E.; Engel, J. A., Ghrelin receptor antagonism attenuates nicotine-induced locomotor stimulation, accumbal dopamine release and conditioned place preference in mice. *Drug Alcohol Depend* **2011,** 117, (2-3), 126-31.
23. Wellman, P. J.; Clifford, P. S.; Rodriguez, J.; Hughes, S.; Eitan, S.; Brunel, L.; Fehrentz, J. A.; Martinez, J., Pharmacologic antagonism of ghrelin receptors attenuates development of nicotine induced locomotor sensitization in rats. *Regulatory peptides* **2011,** 172, (1-3), 77-80.
24. Jerlhag, E.; Egecioglu, E.; Dickson, S. L.; Engel, J. A., Ghrelin receptor antagonism attenuates cocaine- and amphetamine-induced locomotor stimulation, accumbal dopamine release, and conditioned place preference. *Psychopharmacology (Berl)* **2010,** 211, (4), 415-22.
25. Suchankova, P.; Engel, J. A.; Jerlhag, E., Sub-chronic Ghrelin Receptor Blockade Attenuates Alcohol- and Amphetamine-Induced Locomotor Stimulation in Mice. *Alcohol and alcoholism* **2016,** 51, (2), 121-7.

26. Edvardsson, C. E.; Vestlund, J.; Jerlhag, E., A ghrelin receptor antagonist reduces the ability of ghrelin, alcohol or amphetamine to induce a dopamine release in the ventral tegmental area and in nucleus accumbens shell in rats. *European journal of pharmacology* **2021,** 899, 174039.
27. Clifford, P. S.; Rodriguez, J.; Schul, D.; Hughes, S.; Kniffin, T.; Hart, N.; Eitan, S.; Brunel, L.; Fehrentz, J. A.; Martinez, J.; Wellman, P. J., Attenuation of cocaine-induced locomotor sensitization in rats sustaining genetic or pharmacologic antagonism of ghrelin receptors. *Addict Biol* **2012,** 17, (6), 956-63.
28. Havlickova, T.; Charalambous, C.; Lapka, M.; Puskina, N.; Jerabek, P.; Sustkova-Fiserova, M., Ghrelin Receptor Antagonism of Methamphetamine-Induced Conditioned Place Preference and Intravenous Self-Administration in Rats. *International journal of molecular sciences* **2018,** 19, (10).
29. Wenthur, C. J.; Gautam, R.; Zhou, B.; Vendruscolo, L. F.; Leggio, L.; Janda, K. D., Ghrelin Receptor Influence on Cocaine Reward is Not Directly Dependent on Peripheral Acyl-Ghrelin. *Sci Rep* **2019,** 9, (1), 1841.
30. Sustkova-Fiserova, M.; Jerabek, P.; Havlickova, T.; Kacer, P.; Krsiak, M., Ghrelin receptor antagonism of morphine-induced accumbens dopamine release and behavioral stimulation in rats. *Psychopharmacology (Berl)* **2014,** 231, (14), 2899-908.
31. Sustkova-Fiserova, M.; Puskina, N.; Havlickova, T.; Lapka, M.; Syslova, K.; Pohorala, V.; Charalambous, C., Ghrelin receptor antagonism of fentanyl-induced conditioned place preference, intravenous self-administration, and dopamine release in the nucleus accumbens in rats. *Addict Biol* **2019**, e12845.
32. Engel, J. A.; Nylander, I.; Jerlhag, E., A ghrelin receptor (GHS-R1A) antagonist attenuates the rewarding properties of morphine and increases opioid peptide levels in reward areas in mice. *European neuropsychopharmacology : the journal of the European College of Neuropsychopharmacology* **2015,** 25, (12), 2364-71.
33. Sustkova-Fiserova, M.; Jerabek, P.; Havlickova, T.; Syslova, K.; Kacer, P., Ghrelin and endocannabinoids participation in morphine-induced effects in the rat nucleus accumbens. *Psychopharmacology (Berl)* **2016,** 233, (3), 469-84.
34. Sustkova-Fiserova, M.; Charalambous, C.; Havlickova, T.; Lapka, M.; Jerabek, P.; Puskina, N.; Syslova, K., Alterations in Rat Accumbens Endocannabinoid and GABA Content during Fentanyl Treatment: The Role of Ghrelin. *International journal of molecular sciences* **2017,** 18, (11).
35. Jerabek, P.; Havlickova, T.; Puskina, N.; Charalambous, C.; Lapka, M.; Kacer, P.; Sustkova-Fiserova, M., Ghrelin receptor antagonism of morphine-induced conditioned place preference and behavioral and accumbens dopaminergic sensitization in rats. *Neurochemistry international* **2017,** 110, 101-113.
36. Zhao, J.; Du, X.; Chen, M.; Zhu, S., Growth Hormone Secretagogue Receptor 1A Antagonist JMV2959 Effectively Prevents Morphine Memory Reconsolidation and Relapse. *Front Pharmacol* **2021,** 12, 718615.
37. D'Cunha, T. M.; Chisholm, A.; Hryhorczuk, C.; Fulton, S.; Shalev, U., A role for leptin and ghrelin in the augmentation of heroin seeking induced by chronic food restriction. *Psychopharmacology (Berl)* **2020,** 237, (3), 787-800.
38. Charalambous, C.; Lapka, M.; Havlickova, T.; Syslova, K.; Sustkova-Fiserova, M., Alterations in rat accumbens dopamine, endocannabinoids and GABA content during WIN55,212-2 treatment: the role of ghrelin. *International journal of molecular sciences* **2021,** 22, 210.
39. Abizaid, A.; Mineur, Y. S.; Roth, R. H.; Elsworth, J. D.; Sleeman, M. W.; Picciotto, M. R.; Horvath, T. L., Reduced locomotor responses to cocaine in ghrelin-deficient mice. *Neuroscience* **2011,** 192, 500-6.
40. Landgren, S.; Jerlhag, E.; Hallman, J.; Oreland, L.; Lissner, L.; Strandhagen, E.; Thelle, D. S.; Zetterberg, H.; Blennow, K.; Engel, J. A., Genetic variation of the ghrelin signaling system in

females with severe alcohol dependence. *Alcoholism, clinical and experimental research* **2010,** 34, (9), 1519-24.
41. Suchankova, P.; Nilsson, S.; von der Pahlen, B.; Santtila, P.; Sandnabba, K.; Johansson, A.; Jern, P.; Engel, J. A.; Jerlhag, E., Genetic variation of the growth hormone secretagogue receptor gene is associated with alcohol use disorders identification test scores and smoking. *Addict Biol* **2016,** 21, (2), 481-8.
42. Yoon, S. J.; Pae, C. U.; Lee, H.; Choi, B.; Kim, T. S.; Lyoo, I. K.; Kwon, D. H.; Kim, D. J., Ghrelin precursor gene polymorphism and methamphetamine dependence in the Korean population. *Neuroscience research* **2005,** 53, (4), 391-5.
43. Suchankova, P.; Jerlhag, E.; Jayaram-Lindstrom, N.; Nilsson, S.; Toren, K.; Rosengren, A.; Engel, J. A.; Franck, J., Genetic variation of the ghrelin signalling system in individuals with amphetamine dependence. *PloS one* **2013,** 8, (4), e61242.
44. Kroemer, N. B.; Wuttig, F.; Bidlingmaier, M.; Zimmermann, U. S.; Smolka, M. N., Nicotine enhances modulation of food-cue reactivity by leptin and ghrelin in the ventromedial prefrontal cortex. *Addict Biol* **2015,** 20, (4), 832-44.
45. Pilhatsch, M.; Scheuing, H.; Kroemer, N.; Kobiella, A.; Bidlingmaier, M.; Farger, G.; Smolka, M. N.; Zimmermann, U. S., Nicotine administration in healthy non-smokers reduces appetite but does not alter plasma ghrelin. *Hum Psychopharmacol* **2014,** 29, (4), 384-7.
46. Kaabi, Y. A.; Khalifa, M. A., Acute one-cigarette smoking decreases ghrelin hormone in saliva: a pilot study. *Int J Endocrinol* **2014,** 2014, 575671.
47. Kokkinos, A.; Tentolouris, N.; Kyriakaki, E.; Argyrakopoulou, G.; Doupis, J.; Psallas, M.; Kyriaki, D.; Katsilambros, N., Differentiation in the short- and long-term effects of smoking on plasma total ghrelin concentrations between male nonsmokers and habitual smokers. *Metabolism* **2007,** 56, (4), 523-7.
48. Bouros, D.; Tzouvelekis, A.; Anevlavis, S.; Doris, M.; Tryfon, S.; Froudarakis, M.; Zournatzi, V.; Kukuvitis, A., Smoking acutely increases plasma ghrelin concentrations. *Clin Chem* **2006,** 52, (4), 777-8.
49. Wittekind, D. A.; Kratzsch, J.; Mergl, R.; Enzenbach, C.; Witte, V.; Villringer, A.; Kluge, M., Higher fasting ghrelin serum levels in active smokers than in former and never-smokers. *World J Biol Psychiatry* **2020,** 21, (10), 748-756.
50. Bouhours-Nouet, N.; Boux de Casson, F.; Rouleau, S.; Douay, O.; Mathieu, E.; Bouderlique, C.; Gillard, P.; Limal, J. M.; Descamps, P.; Coutant, R., Maternal and cord blood ghrelin in the pregnancies of smoking mothers: possible markers of nutrient availability for the fetus. *Horm Res* **2006,** 66, (1), 6-12.
51. Poykko, S. M.; Kellokoski, E.; Ukkola, O.; Kauma, H.; Paivansalo, M.; Kesaniemi, Y. A.; Horkko, S., Plasma ghrelin concentrations are positively associated with carotid artery atherosclerosis in males. *J Intern Med* **2006,** 260, (1), 43-52.
52. Koopmann, A.; Bez, J.; Lemenager, T.; Hermann, D.; Dinter, C.; Reinhard, I.; Hoffmann, H.; Wiedemann, K.; Winterer, G.; Kiefer, F., Effects of Cigarette Smoking on Plasma Concentration of the Appetite-Regulating Peptide Ghrelin. *Ann Nutr Metab* **2015,** 66, (2-3), 155-161.
53. Fagerberg, B.; Hulten, L. M.; Hulthe, J., Plasma ghrelin, body fat, insulin resistance, and smoking in clinically healthy men: the atherosclerosis and insulin resistance study. *Metabolism* **2003,** 52, (11), 1460-3.
54. Langenberg, C.; Bergstrom, J.; Laughlin, G. A.; Barrett-Connor, E., Ghrelin and the metabolic syndrome in older adults. *J Clin Endocrinol Metab* **2005,** 90, (12), 6448-53.
55. al'Absi, M.; DeAngelis, B.; Nakajima, M.; Hatsukami, D.; Allen, S., Early life adversity and appetite hormones: The effects of smoking status, nicotine withdrawal, and relapse on ghrelin and peptide YY during smoking cessation. *Addict Behav* **2021,** 118, 106866.
56. al'Absi, M.; Lemieux, A.; Nakajima, M., Peptide YY and ghrelin predict craving and risk for relapse in abstinent smokers. *Psychoneuroendocrinology* **2014,** 49, 253-9.

57. Lemieux, A. M.; al'Absi, M., Changes in circulating peptide YY and ghrelin are associated with early smoking relapse. *Biological psychology* **2018,** 131, 43-48.
58. Lee, H.; Joe, K. H.; Kim, W.; Park, J.; Lee, D. H.; Sung, K. W.; Kim, D. J., Increased leptin and decreased ghrelin level after smoking cessation. *Neuroscience letters* **2006,** 409, (1), 47-51.
59. Sahin, S.; Yuce, M.; Alacam, H.; Karabekiroglu, K.; Say, G. N.; Salis, O., Effect of methylphenidate treatment on appetite and levels of leptin, ghrelin, adiponectin, and brain-derived neurotrophic factor in children and adolescents with attention deficit and hyperactivity disorder. *Int J Psychiatry Clin Pract* **2014,** 18, (4), 280-7.
60. Bouhlal, S.; Ellefsen, K. N.; Sheskier, M. B.; Singley, E.; Pirard, S.; Gorelick, D. A.; Huestis, M. A.; Leggio, L., Acute effects of intravenous cocaine administration on serum concentrations of ghrelin, amylin, glucagon-like peptide-1, insulin, leptin and peptide YY and relationships with cardiorespiratory and subjective responses. *Drug Alcohol Depend* **2017,** 180, 68-75.
61. Kara, H., Erdogan, A., Akbas, H., Kuloglu, M.M., The Relationship of Serum Leptin and Ghrelin Levels with Craving and Withdrawal in Opioid Use Disorder. *Alpha Psychiatry* **2021,** 22, (4), 200-205.
62. Riggs, P. K.; Vaida, F.; Rossi, S. S.; Sorkin, L. S.; Gouaux, B.; Grant, I.; Ellis, R. J., A pilot study of the effects of cannabis on appetite hormones in HIV-infected adult men. *Brain research* **2012,** 1431, 46-52.
63. Farokhnia, M.; Grodin, E. N.; Lee, M. R.; Oot, E. N.; Blackburn, A. N.; Stangl, B. L.; Schwandt, M. L.; Farinelli, L. A.; Momenan, R.; Ramchandani, V. A.; Leggio, L., Exogenous ghrelin administration increases alcohol self-administration and modulates brain functional activity in heavy-drinking alcohol-dependent individuals. *Molecular psychiatry* **2017**.
64. Farokhnia, M.; McDiarmid, G. R.; Newmeyer, M. N.; Munjal, V.; Abulseoud, O. A.; Huestis, M. A.; Leggio, L., Effects of oral, smoked, and vaporized cannabis on endocrine pathways related to appetite and metabolism: a randomized, double-blind, placebo-controlled, human laboratory study. *Translational psychiatry* **2020,** 10, (1), 71.
